# An Overview on Food Applications of the Instant Controlled Pressure-Drop Technology, an Innovative High Pressure-Short Time Process

**DOI:** 10.3390/molecules26216519

**Published:** 2021-10-28

**Authors:** Juan Leopoldo Pech-Almeida, Carmen Téllez-Pérez, Maritza Alonzo-Macías, Giselle Dení Teresa-Martínez, Karim Allaf, Tamara Allaf, Anaberta Cardador-Martínez

**Affiliations:** 1 Tecnologico de Monterrey, Escuela de Ingeniería y Ciencias, Epigmenio González 500, San Pablo, Querétaro 76130, Mexico; juanlpecha@gmail.com (J.L.P.-A.); ctellezperez@gmail.com (C.T.-P.); malonzoma@tec.mx (M.A.-M.); gis_teresa20@outlook.com (G.D.T.-M.); 2 Laboratory of Engineering Science for Environment LaSIE-UMR-CNRS 7356, Eco-Intensification of Agro-Industrial Eco-Processes, La Rochelle University, 17042 La Rochelle, France; kallaf@univ-lr.fr; 3 ABCAR-DIC Process, 17000 La Rochelle, France; tamara.allaf@abcar-dic.com

**Keywords:** swell-drying, extraction, decontamination

## Abstract

Food processing systematically aims at meeting the needs of consumers who are looking for total high quality and perfect food safety. As the various thermal and non-thermal food preservation technologies often affect the natural properties in terms of sensation, flavor, texture, etc., instant controlled pressure drop (DIC) has been conceived as a relevant, innovative process in this field. DIC uses high saturated steam pressure and short duration to provide a new way to expand biological matrices, improve drying, decontaminate, and extract biologically active compounds, among other attributes. Therefore, this review focuses on describing the applications of DIC technology on a wide range of products such as foods and by-products that have been processed both in the laboratory and on an industrial scale. The application of DIC has shown the possibility of a significant leap in quality improvement and cost reduction in the food industry. DIC reduces the drying time of fruits and vegetables, and improves the extraction of essential oils, vegetable oils, and antioxidant components. It also provides strong decontamination, eliminates vegetative microorganisms and spores, and reduces non-nutritional and allergenic components. Over the past 33 years, this technology has continued to expand its food applications and improve its characteristics on an industrial scale. But there are still many food unit operations that can be taken to the next level with DIC.

## 1. Introduction

The “instant controlled pressure-drop technology”, known by its French acronym DIC (Détente Instantanée Contrôlée), was created in 1988 as a solution to the drying shrinkage/collapse issues, in order to obtain a better quality of the dried plants in terms of texture, color, aroma… [1]. Since then, the DIC technology has led to remarkable developments in drying, extraction, microbial decontamination, allergenicity reduction, deodorization of vegetable oils, desolvation, etc., of plants and various foods.

DIC is a thermo-mechanical process based on the theory of instantaneous thermodynamics, applied to heat-sensitive products via treatments of high temperature/high pressure for a short time. Thus, the DIC process consists of subjecting biological food matrices to saturated steam pressure treatments of 100 to 900 kPa for a few seconds, followed by an abrupt and controlled pressure-drop at a rate higher than 500 kPa per second; this leads to a final vacuum of absolute pressure of 10 to 5 kPa, significantly lower than 101.325 kPa (which is the atmospheric pressure at sea level). The instant controlled pressure-drop is at the heart of the DIC technology, as it triggers instantaneous autovaporization of water, quick cooling of biological products, and expansion and creation of cells in the matrix [1,2]. Indeed, from a situation very far from the equilibrium in temperature and especially in vapor pressure, the system immediately evolves by instant autovaporization and brings a quick expansion and an abrupt cooling of biological matrices. It also engages rapid expulsion of adequate molecules, which would significantly improve food processing [3].

**Figure 1:**
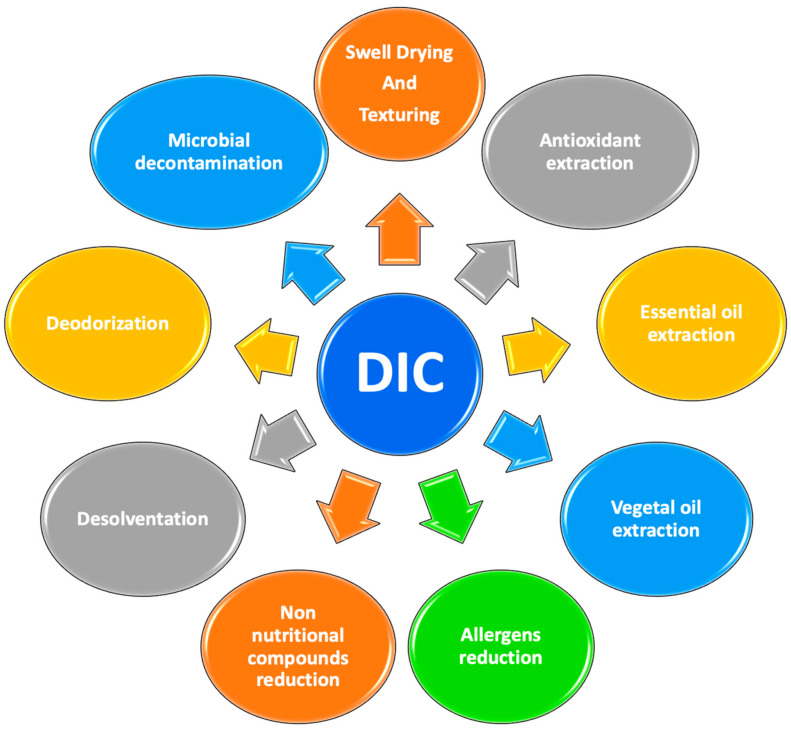
Main applications of the instant controlled pressure-drop DIC technology in food processing.

In drying operations, DIC provides a new way to expand biological matrices. It reduces energy consumption and improves the overall quality of dried products [4]. DIC improves extraction kinetics by providing better solvent penetration and leaching or solubilization of molecules in the extraction operation. In addition, it improves the overall quality of the final products by reducing thermal degradation [5,6]. Moreover, thanks to the thermo-mechanical effect, DIC guarantees results of great relevance of elimination/destruction of microorganisms in both vegetative and spore forms [7,8]. Currently, the study of the performance of DIC aims at the reduction or even elimination of food mycotoxins. Moreover, in purification operations, DIC is effective in the deodorization and desolvation processes [9,10], with interesting results on reducing non-nutritional and allergenic molecules [11,12]. Figure 1 summarizes the main applications of instant controlled pressure-drop technology in food processing.

## 2. Fundamentals of the Instant Controlled Pressure-Drop Technology

**Figure 2:**
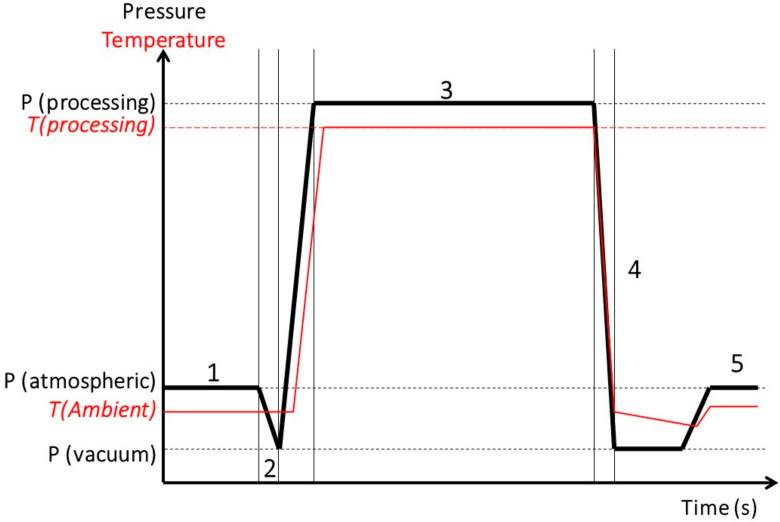
Typical pressure/temperature-time profile for a DIC processing cycle: (1) introduction of food material to the reactor; (2) establishment of an initial vacuum in the processing reactor; (3) injection of dry saturated steam until achieving and retaining a previous target pressure during a selected treatment time; (4) instant controlled pressure-drop towards a vacuum, and (5) releasing to atmospheric pressure.

The DIC treatment is described as a five-step thermo-mechanical treatment. Figure 2 shows the five steps of one DIC treatment cycle through a schematic time-pressure/temperature graph. (1) The initial stage consists of introducing the food material into the DIC reactor at atmospheric pressure; (2) the vacuum establishment stage creates a final absolute pressure of 10 to 5 kPa into the DIC reactor to ensure better performance of the subsequent step; (3) the hydrothermal processing stage injects saturated steam into the DIC reactor until achieving and retaining a previous target pressure during a defined treatment time; (4) the abrupt pressure-drop stage involves carrying inside the DIC reactor an instant controlled pressure-drop towards a vacuum (between 10 to 5 kPa); and (5) the atmospheric releasing stage consists of re-establishing the atmospheric pressure inside the DIC, usually using impingements on the product surface. It is worth highlighting that according to the characteristics of the biological matrices and the objectives pursued, it could be possible to apply more than one DIC cycle. Then, steps 2 through 4 are repeated up to the required number of cycles.

**Figure 3:**
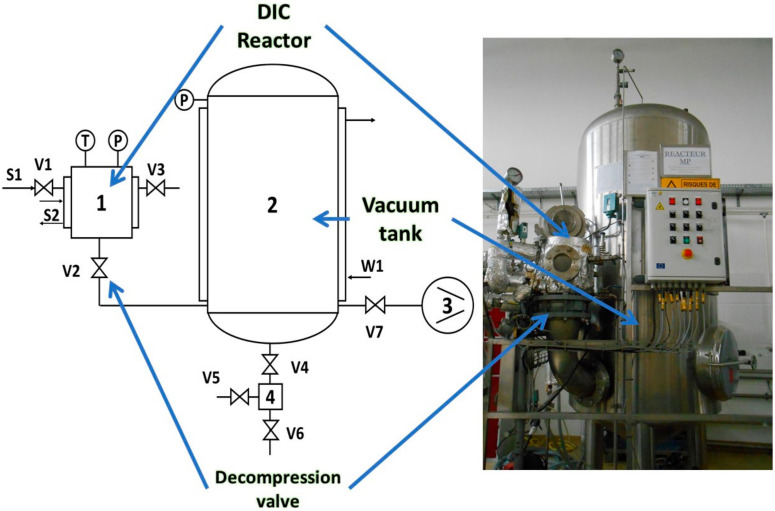
Left: Schematic diagram of DIC Equipment: (1) DIC reactor; (2) vacuum tank; (3) vacuum pump; (4) Trap, V1-V7-valves, S1, and S2 saturated steam injection, W1- cooling water. Right: Laboratory DIC equipment (ABCAR-DIC Process, Compiègne, France).

DIC processing of foods involves many operating parameters, which are perfectly controlled in the DIC equipment supplied by ABCAR-DIC Process (Compiègne, France). Figure 3 shows the schematic of a laboratory DIC unit. There are two main components: the housing vessel and the vacuum tank. The treatment chamber is particularly integrative because it is where all the thermal and mechanical transformations occur [13]. Depending on the application, dry saturated steam, supersaturated steam, or other gases can be used as the simultaneous heat transfer medium and pressure increases. Generally, in the case of food, dry saturated steam is used, with pressure ranging from 100 kPa (99.61 °C) to 700 kPa (164.95 °C). The process vessel is mainly connected to a saturated steam generator, a vacuum tank, and the atmosphere. The whole equipment allows for the measuring, controlling, and recording of the pressure changes [3,14]. The vacuum component acts in stages 2 and 4, and it is mainly formed by a double-shell vacuum tank, a vacuum pump, and a cooling system. The instantaneous valve connects it to the processing vessel. The previously established vacuum becomes essential in the steam injection step as it improves the exchange between the steam and the effective surface of the product [13]. The abrupt drop towards the vacuum creates mechanical stress in the micropores of the biological matrix mainly due to the generated steam. The necessary amount of heat to be lost during the transition from the treatment temperature level to the equilibrium temperature level defined by the new vacuum level involves the phenomenon called “autovaporization”. In step 4, instantaneousness is essential to achieve the instant pressure drop whose rate is at least 0.5 MPa/s [3]. The final puff is highly dependent on the pressure in the vacuum tank. In addition, cooling is greater at a higher vacuum and protects the biological matrix from the undesirable heat-derived effect. In general, the highest pressure in the vacuum vessel is between 5 and 10 kPa [4].

DIC technology is also currently applied at research and industrial levels. Depending on the application, products can be recovered from the treatment vessel, while extracts and liquids can be collected from the vacuum tank. Moreover, it is important to highlight that the processing conditions defined at laboratory scale can be directly applied at industrial scale, which becomes a further advantage of DIC technology.

Since 1988, research efforts at laboratory and industrial scales have allowed the development of numerous applications of the DIC technology on food processing. In fact, by reviewing the Scopus database under the keyword “Instant controlled pressure-drop”, nearly two hundred scientific articles show that DIC is a greatly relevant innovative technology able to highly intensify various food unit operations. In this respect, the main food applications of the instant controlled pressure-drop technology will be detailed hereafter.

## 3. Instant Controlled Pressure-Drop Process on Food Drying

Thanks to its ease of implementation, great convenience, high efficiency with a long shelf life and a vast range of products, extensive possibilities of equipment and treatment modes, drying is one of the most used preservation processes. Among all the industrial food drying operations, convective air drying (CAD) is one of the most applied. However, it presents some drawbacks, including an inevitable shrinkage and collapse of the product structure, low kinetics, organoleptic and nutritional losses due to the long drying periods, and in many times microbial contamination [15]. Then, to enhance the CAD, the instant controlled pressure-drop technology has performed excellent results to improve the drying process and the total quality of dried products.

Since 1988, existing studies of DIC treatment coupled to convective air drying have shown that through a well-controlled modification of the product texture, it is possible to address the shrinkage, which is the most awkward and key problematic aspect of conventional drying of food [15,16]. This coupling has been named “Swell-drying”, which involves an airflow drying stage couple to a DIC texturing stage [15]. A swell-drying operation generally consists of a first airflow pre-drying stage until a specific water content (most of the times between 0.20 g and 0.50 g H_2_O/g of solid); followed by a DIC texturing stage (100–900 kPa during some seconds, followed by an abrupt controlled pressure-drop); and a final air-drying stage (generally until a final water content of around 0.05 g H_2_O/g of solid). In most fruit and vegetables, the pre-drying stage is concluded when it extends a level of water content that allows the product to reach the glass transition due to the DIC pressure-drop, thus avoiding future collapse [15]. However, this stage is not mandatory, and according to expected results, DIC operation can also be applied directly to fresh food (e.g., in onion [17] and chicken meat [18]).

To be able to evaluate the impact of DIC treatment on the intensification of the food drying process, it is required to study some aspects, such as (1) the structure and main characteristics of food polymers, (2) the process performance in terms of kinetics and energy consumption, and (3) the quality attributes of the final products.

### 3.1. Impact of DIC Treatment on Fruits and Vegetable Drying

Most fresh fruit and vegetables are composed of around 70 to 95% water [19]. Consequently, during hot air drying, these products lose their original volume, and their cells collapse. As the natural structure of fruit and vegetables tends to be compact, their water permeability through the cell wall and cell-organized matrix tends to weaken. This phenomenon triggers low values of effective global diffusivity, resulting in low kinetics of both drying and rehydration.

**Figure 4:**
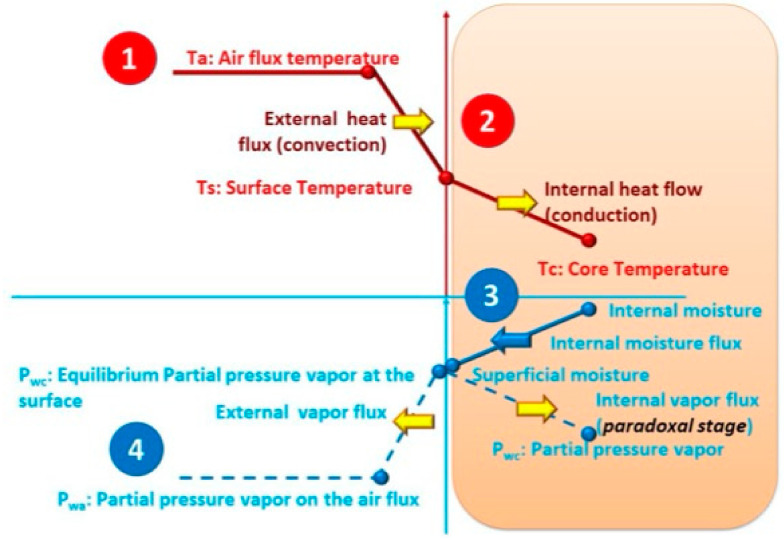
Scheme of the main transfer phenomena during convective airflow drying. (1) Heat transfer by convection; (2) Heat transfer by conduction within the food matrix; (3) Water transfer by diffusion and (4) Mass transfer by evaporation. Modified from Allaf et al. [3].

According to Allaf et al. [16], after a complete fundamental analysis of the driving forces and resistances occurring during the convective airflow drying operation (CAD), three main stages arise: (1) the starting accessibility by airflow washing and purely superficial evaporation, (2) the diffusion of liquid water within the matrix to evaporate at the exchange surface, and (3) the paradoxical stage of internal heat and vapor transfers within the matrix. Figure 4 shows a schematic diagram of heat and mass transfer phenomena occurring during CAD. Moreover, for perfectly intensified external airflow conditions, the effective diffusivity of water within the matrix is the limiting phenomenon of the drying process′s main stage (Stage 2).

The first stage implies mass and heat convection transports from the interaction surface towards the surrounding medium. In this short time stage, the interaction between airflow and the product surface triggers superficial dehydration. The higher the airflow velocity, the more intense the dehydration without any limiting value of airflow velocity [20]. Thus, the drying ratio during this short stage named the starting accessibility is defined as the amount of water directly lost by the product′s surface before starting any diffusion mechanism within the product [3].

In the second stage of drying, five mass and heat transfer phenomena have been identified: (1) the heat transfer from the airflow towards the interaction surface by convection; (2) the heat diffusion from the surface toward the core of the material by conduction; (3) the diffusion of liquid water within the porous medium from the core to the surface; (4) the generation of vapor from the water interacting with the airflow at the surface; and (5) the transport of vapor towards the external medium far from the surface. At this point, by guaranteeing high airflow temperature and velocity, with low relative humidity and adequate interaction surface, the external resistance of vapor transport is made so negligible that water diffusion [4] becomes the limiting phenomenon. Moreover, during the main stage of airflow drying, the shrinkage phenomenon implies an apparent fall of effective diffusivity.

The third stage occurs when the transfer of water occurs exclusively in the vapor phase. When water activity is constant, the vapor pressure is higher at the surface than in the internal part of the matrix. This phenomenon triggers a paradoxical state because drying takes place through “front progression” kinetics [3].

During CAD, there is some resistance to water flux; however, the DIC technology can solve all of these difficulties. Thanks to the expansion of the internal pores generated by the instant autovaporization of residual water after the pre-drying stage, DIC leads to the recovery of the original volume of pre-dried fruit and vegetables. Moreover, this texture change has significantly improved the post-drying kinetics of these products, and it has also allowed better preservation of bioactive molecules and decontamination.

This section presents the main findings of the impact of DIC technology on fruit and vegetable drying.

#### 3.1.1. Instant Controlled Pressure-Drop Treatment on Fruits

One of the most studied swell-drying fruits has been apple (*Malus domestica*) [21,22,23,24,25,26]. Generally, the initial water content of this fruit ranges from 4 to 7 g H_2_O/g db (dry basis) (80% to 87.5% wet basis). Then, to achieve a final water content of 0.04 g H_2_O/g db, the study of Mounir et al. [27] divided the total swell-drying operation into three steps. First, a CAD pre-drying stage to reach a water content of 0.14 g H_2_O/g db, followed by a DIC texturing stage, and a final CAD drying stage. DIC textured samples had a significantly quicker post-drying stage from 0.14 to 0.04 g H_2_O/g db, which only required one h, instead of 6 h for non-textured samples. Moreover, under a DIC treatment of 300 kPa and 80 s, a significant increase of quercetin was reached, and was found to be 500%–700% more than the initial amount before treatment.

On the other hand, Li et al. [25] studied the mechanism of DIC treatment to develop apple cubes with a crisp texture. They mainly focus on the correlation between the water content of samples after the pre-drying stage and the performance of DIC to generate expansion. Their study indicated that the highest expansion of apple cubes was obtained under pre-dried samples at a water content ranging between 0.134–0.248 g H_2_O/g db. They also highlighted that a good expansion effect of DIC texturing could be achieved when samples cross the rubber behavior to a vitreous behavior during DIC decompression.

Xiao et al. [28] studied the effects of DIC texturing on the characteristics of cell wall polysaccharides of apple slices and their relationship to the texture (Table 1). In this study, apple samples were pre-dried until a water content of 0.3 g H_2_O/g db, then textured by DIC, and finally dried by continuous vacuum drying. Obtained results showed that it is possible to get apple chips with a crisp texture and excellent honeycomb-like structure by coupling CAD to the DIC texturing treatment. Furthermore, swell-dried samples showed an excellent rehydration ratio thanks to a homogenous porous structure and a large specific surface area. Moreover, concerning fresh apples, CAD and swell-dried apples exhibited a decrease in water-extractable pectin fraction, which according to the authors might be partially attributed to the depolymerization and leaching of the pectic polysaccharides.

In addition, to modify texture, DIC has also produced a decrease in polyphenol oxidase activity (PPO activity), which is the enzyme responsible for browning. Gao et al. [21] studied the effect of swell-drying in apple slice browning and proved that coupled CAD and DIC contributed to a decrease in the PPO activity of fresh apples. These results were attributed to the high temperature of DIC treatment, which could inactivate this enzyme. However, apple browning is also related to the high content of sugars and the Maillard reaction products generated during drying; even if PPO activity is eliminated, special attention is needed to select the DIC texturing conditions to avoid browning.

On the other hand, Li et al. [24] showed that for accelerating the water diffusion during pre-drying apple cubes, hydrolysis by pectinase and pectin osmosis pre-treatments could be applied. However, those pre-treatments can affect the internal pore diameter and wall thickness, directly impacting the final texture. Then, to preserve the crispness of DIC dried apple cubes, the authors emphasize using short-time and low concentration pectin osmosis treatments. In this respect, Li et al. [23] underline that an osmotic pre-treatment with maltodextrin or microcrystalline cellulose before pre-drying allowed increasing crispness and volume (1.6 times) for CAD samples.

Additionally, DIC texturing has also been applied on the dehydrofreezing of apple slices. Dehydrofreezing is a modification of freezing to reduce tissue damage and drip loss in thawing, in which food is previously dehydrated to a desirable water content, and then it is frozen [41]. In this respect, Ben Haj Said et al. [32] studied the impact on apple slices′ firmness. Samples were submitted to a pre-drying through CAD, followed by a DIC texturing treatment and final freezing. The initial water content of fresh apples was 7 g H_2_O/g db, and samples were pre-dried at different water content values ranging from 0.30 to 2.0 g H_2_O/g db, followed by different DIC treatments. Consequently, the DIC operative parameter influencing apple firmness was the initial water content, 1.66 g H_2_O/g db being identified as the critical water content value.

Concerning sorption isotherms of swell-dried apples, Iguedjtal et al. [22] observed that both CAD dried and swell-dried apples presented a type III isotherm profile and also that the DIC texturing treatment allowed increasing the specific surface area of swell-dried apples.

On the other hand, Setyopratomo et al. [34] studied the effect of swell-drying on the dehydration kinetics, water and oil holding capacity, and nutritional characteristics of bananas (*Musa paradisiaca*) (Table 1). First, fresh bananas were cut into 16 × 16 × 2 mm pieces and pre-dried under CAD at 50 °C until 0.25 g H_2_O/g db. After that, they were submitted to a DIC texturing treatment, a post-drying at 70 °C until 0.075 g H_2_O/g db, and a grinding. Drying kinetics showed that DIC texturing increased the effective water diffusivity by 23%, from 8.11 to 10.01 × 10^−10^ m^2^/s. Moreover, the statistical analysis showed that the water holding capacity increased 290% under high-pressure conditions under DIC treatments, reaching 7.8 g H_2_O/g db against 2.0 g H_2_O/g db concerning the non-textured samples. This was not the same for the oil holding capacity, where swell-dried samples performed 0.60 g oil/g db against 1.30 g oil/g db for non-textured samples. Finally, it is worth noting that DIC treatment inhibited the transformation of banana starch to reduced sugar.

Strawberry (*Fragaria vesca var. vesca*) is also an important fruit worldwide. Swell-drying has shown to be an excellent way to develop strawberry snacks in less time than CAD, with a high crispness behavior and a high rehydration capacity. The first study about the effect of pressure, time, and water content on DIC texturing of strawberries was carried out by Nouviaire et al. [35]. The fresh strawberries were cut manually into pieces of 2 cm thickness, and they were pre-dried under CAD at 50 °C until a water content range between 0.05 to 0.20 g H_2_O/g db. After that, they were submitted to a DIC texturing treatment and a post-drying at 50 °C until 0.05 g H_2_O/g db. Obtained results showed that DIC increases the expansion rate of dried fruit up to 2.4 times that of the non-treated samples, while it reduces the drying time up to 63%. Moreover, this study highlighted the importance of a good selection of DIC texturing parameters, indicating that the optimal pressure conditions for swell-dried strawberries with a crisp texture were between 220 and 350 kPa.

Following this line of research, Alonzo-Macías et al. [37] studied the macro (porosity and relative density) and micro-structure (cell characteristics) of swell-dried strawberries. Compared to CAD, DIC swell-dried strawberries were threefold more expanded. Additionally, through a puncture test, they identified a high crispness behavior on DIC samples. Crispness was defined as small continuous linear elastic behavior up to the successive fractures during chewing, then the swelled products performed a significant number of micro-ruptures, with weak individual rupture force and work. Since crispness is an essential sensory attribute for snack products, swell-drying efficiently addresses consumer requirements. Maritza et al. [38] also showed that DIC significantly impacts strawberries′ drying and rehydration kinetics, increasing the effective diffusivity and the starting accessibility compared to CAD. Moreover, Alonzo-Macías et al. [36] showed that the optimal conditions of DIC to obtain the highest levels of phenols, flavonoids, and anthocyanins and antioxidant activities were 350 kPa for 10 s (Table 1). Thus, DIC allowed the development of strawberry snacks in less time than CAD, with a high crispness behavior and a high bioactive molecule preservation.

Berrycactus (*Myrtillocactus geometrizans*) is a perennial Cactaceae plant native from Mexico which produces dark purple berries locally called “garambullo”. Santiago-Mora et al. [39] studied the effect of freeze-drying and swell-drying on preserving in-vitro antioxidant capacity and bioactive compounds of ripe berrycacti. Before DIC treatment, fruits were pre-dried at different water content (0.10, 0.14, 0.20, 0.26, and 0.30 g H_2_O/g db). DIC treatments were carried out between 100 to 450 kPa and from 5 to 45 s. Both drying methods demonstrated high antioxidant activity, highlighting the increase of extractable polyphenols and condensed tannins and good retention of ascorbic acid and betalains after the drying treatments. The optimal DIC conditions for the highest antioxidant capacity were a water content of 0.20 g H_2_O/g db, a saturated steam pressure of 450 kPa, and a processing time of 25 s (Table 1).

Swell-drying has also been applied to develop ready-to-eat snacks and expanded granule powders from Zaghloul dates (*Phoenix dactylifera* L.) [40]. Dates were pre-dried under CAD until a water content of 0.12 g H_2_O/g db was reached to be DIC textured at different operative parameters (200 to 600 kPa and from 9 to 35 s). The quality attributes of these snacks and expanded granule powders were evaluated in terms of texture, color, and sensory characteristics. DIC treatment of 600 kPa and 22 s (Table 1) allowed an expansion ratio of 146%. However, the best color corresponded to 200 kPa and 22 s. Likewise, the hardness, springiness, cohesiveness, and chewiness of swell-dried dates were lower than CAD.

#### 3.1.2. Instant Controlled Pressure-Drop Treatment on Fruit Byproducts

Sustainability within the food industry requires new technologies to valorize fruit processing by-products. In this respect, the DIC technology has shown exciting results in developing new applications for byproducts such as snacks and innovative ingredients with new physicochemical and techno-functional properties. Introduced below are some examples of DIC effects on fruit by-products (Table 2).

A standard method of consuming cactus pear fruit (*Opuntia ficus-indica*) is to make juice; however, peels are the principal “waste” after juice extraction. Around 38% of fruit weight is the peel. In this respect, Namir et al. [42] applied a DIC treatment on opuntia fruit peels to develop nutritious snacks and powder. Fruit peel was pre-dried to 0.15 g H_2_O/g db, and DIC processing parameters were studied in the ranges of 100 to 600 kPa and 2 to 25 s. The swell-dried peels resulted in high crispness snacks with improved structural and nutritional attributes. The optimal conditions to reach the highest bioavailability of phenolic compounds and β-carotene of pear fruit snacks were 600 kPa and 15 s. Moreover, swell-drying allowed obtaining high porous snacks with a relative expansion ratio of up to 21 times more than CAD, which facilitates the production of high-quality expanded granule powder.

Louati et al. [43] applied the DIC technology on orange by-products (25% peel, 74% pulp, and 1% seeds) to improve drying kinetics and phenolic compounds′ extraction. Orange by-products were pre-dried till 0.42 g H_2_O/g db, and studied. Operative DIC parameters were a saturated steam pressure from 300 to 600 kPa, a treatment time from 20 to 220 s, and 1 to 7 DIC cycles. DIC texturing allowed increasing the effective diffusivity and the starting accessibility, which directly impacts the reduction of total drying time, reducing it from 300 min (non-treated) to 60 min. The optimum value of diffusivity was obtained at 600 kPa, 20 s, and seven cycles. DIC also improved the solvent extraction of hesperidin and rutin by 537% and 236% for raw material. Optimal DIC conditions to improve flavonoids extraction were defined as 490 kPa, 186 s, and five cycles. Further details about the impact of DIC texturing on the extraction processes of essential oils, antioxidants [14,45], and pectin [46] extraction from orange by-products can be viewed in Section 4.

On the other hand, Albitar et al. [44] studied the effect of swell-drying on pomace and seeds issued from cranberry juice processing. First, both byproducts were pre-dried between 0.05 to 0.45 g H_2_O/g db, and they were submitted to DIC texturing under 200–500 kPa for 5 to 15 s. DIC treatment increased the effective diffusivity and the starting accessibility of both byproducts, which means a reduction of total drying time of at least 50%. Moreover, thanks to autovaporization, it was possible to generate expanded granule powders, which means a decrease in explosion risk.

#### 3.1.3. Instant Controlled Pressure-Drop Treatment on Vegetables

The first studies about the impact of DIC texturing on vegetables were carried out on carrots (*Daucus carota*) by Louka et al. [4] and Louka et al. [13]. Dice carrots (16 × 16 × 2 mm^3^) were blanched (5 min in water at 95 °C) and pre-dried at different levels of water content (between 0.03 and 0.40 g H_2_O/g db). After that, they were submitted to a DIC texturing under 200–600 kPa for 2 to 30 s. The impact of DIC treatment was evaluated through the expansion ratio, the color, and the degree of cooking. Results highlighted the importance of various aspects, such as (1) the establishment of an initial vacuum state before the saturated steam injection; (2) the atmospheric air impingement stage, which further reduces the product temperature and fixes the newly expanded state; and (3) the correct selection of water content (W). If W was lower than 0.05% g H_2_O/g db, dried carrots were often caramelized. Moreover, the optimal DIC conditions for expanded dried carrots were identified as 0.25 g H_2_O/g db, 450 kPa, and 25 s. Table 3 summarizes the main findings of DIC impact on vegetables drying.

On the other hand, Nguyen et al. [47] studied the impact of DIC on drying kinetics and some physical properties of dried carrots. First, fresh carrots were washed, peeled, cut into cubes (2 cm), and blanched (1 min in boiling water for 1 min). After that, carrots were dried at 45 °C to a 25.49% dry basis. DIC texturing was carried out under a steam pressure between 100–500 kPa for 5–55 s. Results showed that after the DIC texturing stage, the bulk density was decreased, while the porosity, the absolute expansion, the effective water diffusivity, and the starting accessibility were increased. Optimal DIC conditions to increase the effective diffusivity 3.2 times vs. CAD were identified as 0.2549 g H_2_O/g db, 440 kPa, and 48 s.

The viscoelasticity of food components influences the modification of volume expansion of swell-dried products. To better understand the role of DIC texturing on cell wall pectic polysaccharides of carrots (CWPs), Peng et al. [49] studied the characteristics of CWPs from ground tissue (GT), junction of ground and vascular tissue (JT), and vascular tissue (VT) of swell-dried carrots. This study showed that the characteristics of CWPs significantly affect the expansion ratio and textural properties of swell-dried carrots. Moreover, the most homogeneous pore size distribution was derived from the GT zone.

It must be pointed out that there are two types of DIC equipment at an industrial scale: (1) the DIC equipment developed and widely studied by the research group of Pr. Karim Allaf [3], dominant in the European and American markets, which is characterized by the direct injection of steam into the sample chamber, which means a pressure processing between 100–900 kPa; and (2) the DIC equipment mainly reviewed by Bi and collaborators [29], predominant in the Chinese market, and which is characterized by heat radiation through steam pipes, which means a processing pressure not greater than 100 kPa. This difference in the heating procedure becomes a key factor because the total pressure and temperature gradients are significantly different, directly impacting samples′ expansion and instant autovaporization. For this reason, in the case of Chinese DIC equipment, to improve the texture and porous characteristics of final DIC dried carrots, the effects of some pre-treatments as such as freezing [50] and osmotic dehydration have been studied [48].

Peng et al. [50] studied the effects of four different freezing pre-treatments coupled with osmotic dehydration on DIC dried carrots′ texture and porous characteristics. First, fresh carrots were washed, peeled, and cut (10 × 10 × 40 mm). After that, carrots were frozen at −18 °C, −40 °C, and −80 °C. Osmotic dehydration was carried by a first thawing stage at room temperature (26.5 °C) for 2 h, followed by an immersion into hot sucrose solution (98 ± 2 °C, 40 °Bx) for 20 min. Next, samples were pre-dried in a hot air dryer at 70 °C until a water content of 0.50 g H_2_O/g db, and, after that, they were submitted to a DIC texturing (95 °C for 15 min followed by an instant pressure-drop to vacuum). Finally, carrots were dried at 70 °C for 3–4 h under vacuum drying (average final water content was below 0.05 g H_2_O/g db). Results showed that the formation of ice crystals during freezing at −18 °C and −40 °C could modify the carrot microstructure and thereby enhance the DIC expansion. Besides, regarding the effect of osmotic dehydration pre-treatment on carrots, Peng et al. [48] showed that the samples appropriately pretreated (e.g., 60 °Bx for 4 or 6 min) performed a great overall quality (volume, texture, microstructure, color, and sensory attributes). The expansion of the DIC-dried samples is highly dependent on solute gain.

Finally, Sahyoun et al. [51] also studied the effect of blanching, freezing/thawing, and steaming as pre-treatments before DIC texturing of carrots. Steam blanching was performed at 110 °C for 10, 12, or 30 min. Freezing/thawing was studied under fast, intermediate, slow, and very slow freezing levels. Steaming was carried out inside the DIC reactor at 250, 350, 450, and 550 kPa during 20 s, followed by two types of decompression, progressive decompression requiring two to three min to reach the atmospheric pressure and instantaneous pressure-drop within 200 ms (milliseconds) to reach 5 kPa. After each pre-treatment step, samples were dried by vacuum. The intermediate freezing/thawing pre-treatment and DIC steaming reduced the drying time significantly, from 247 min for the control to 50 min and 71 min, respectively. Long freezing/thawing induced more dilation and cellular breakdown, and thus facilitated the diffusion of water within the cells during dehydration.

Swell-drying has also been studied on potatoes (*Solanum tuberosum*) [1,4,13]. In this case, potatoes (10 × 10 × 2 mm^3^) were blanched (7 min in water at 95 °C) and pre-dried at different levels of water content (between 0.03 and 0.50 g H_2_O/g db). After that, they were submitted to a DIC texturing under 300–700 kPa, for 2 to 45 s. The faster the decompression, the better the dehydration kinetics. The most significant increase in the expansion ratio was observed when the decompression duration lasted between 0.7 and 8 s. Moreover, thanks to DIC texturing, the time required for drying potato pieces from 0.25 to 0.4 H_2_O/g db was reduced from 220 min for the control (CAD) to 100 min for swell-dried samples [1]. Besides, thickness seemed to play a critical role in terms of the expansion ratio, having observed that a thickness of roughly 3 mm could avoid the matter′s resistance to deformation. Optimal DIC conditions for dice potatoes were identified as 0.15 g H_2_O/g db, 700 kPa, and 40 s [13].

Iguedjtal et al. [52] studied the sorption isotherms of swell-dried potatoes subjected to two DIC texturing conditions (300 and 600 kPa, both for 20 s). Before DIC treatment, samples were pre-dried at 0.15 g H_2_O/g db, and CAD at 60 °C was used as control. DIC texturing increased the surface area of potato slices by 45% relative to CAD. The water activity (A_w_) of DIC samples was lower for both treated samples. E.g., for water content of 0.10 g H_2_O/g db, the reported A_w_ for CAD sample was 0.49, and for DIC samples was 0.39 for 300 kPa and 0.28 for 600 kPa. These lowered water activities likely describe a more shelf-stable behavior than CAD materials for the same water content.

Louka et al. [13] also studied the effect of swell-drying on tomatoes (*Solanum lycopersicum*). First, tomatoes were cut (5 × 5 × 10 mm^3^) and pre-dried at a water content of 0.20 g H_2_O/g db. They were then submitted to a DIC texturing under 100–700 kPa for 5 to 45 s. DIC-textured tomatoes were between 2.2 to 4.5 times the volume of CAD′s sample. Optimal DIC conditions to achieve the highest expansion ratio (around 4.5) were identified as 0.20 g H_2_O/g db, 700 kPa, and 15 s. However, under these conditions, samples become overcooked and, once rehydrated, presented with a mushy structure. These characteristics could be crucial for the development of instant food like soups, but not for snacks. On the other hand, under 0.20 g H_2_O/g db, 400 kPa, and 30 s, it was possible to achieve an expansion ratio of around 4, with the firmest structure after rehydration. Besides, González-Sánchez et al. [53] identified that at 12 g H_2_O/g db, 400 kPa, and 25 s, DIC-texturing performed good preservation of the total phenolic compounds and the antioxidant capacity (IC_50_) of tomato slices. One of the distinguishing features of DIC technology is the many treatment parameters that can be chosen to achieve specific physicochemical characteristics.

Dried onion (*Allium cepa*) is a commonly used vegetable for cuisine and culinary preparations; however, CAD products tend to be challenging to rehydrate. For that, the DIC technology has been applied on onions under two conditions: (1) as swell-drying and (2) as a blanching–steaming pre-treatment before CAD due to structural damage.

For swell-drying (SD) onions, two studies have been reported. The first was carried out by Louka et al. [4], in which onions were cut into strips of 5 mm before being pre-dried until a water content between 0.30 and 0.40 g H_2_O/g db was reached. After that, they were submitted to a DIC texturing under 200 to 600 kPa, during 2 to 40 s, followed by a post drying stage. For this study, results showed that swell-dried onions tripled the volume compared to CAD. Optimal DIC conditions to achieve the highest expansion ratio (around 2.8) without overcooking were identified as 0.10 g H_2_O/g db, 450 kPa, and 15 s. Additionally, it was highlighted that swell-dried onion strips change color according to treatment conditions, and they may be white, yellowish, golden, browned, or even caramelized [4]. The second study, by Mounir et al. [27], evaluated the drying kinetics and sorption isotherms of swell-dried onion. For that, fresh onions were cut (50 mm length, 5 mm width, and 2 mm thickness) and pre-dried until a water content of 0.30 g H_2_O/g db. After that, a DIC texturization was carried under 200 to 500 kPa for 5 to 15 s. Finally, treated samples were post-dried (0.05 g H_2_O/g db) and ground. Results showed that DIC improved drying kinetics by reducing the drying time by 78%, from 681 ± 10 min (CAD) to 150 ± 10 min (SD). Optimal DIC conditions to improve drying kinetics were determined under a saturated steam pressure between 350 and 550 kPa and 10 s. Moreover, as onion behavior as anisotropic material, water transfer was performed in one axis (width= 5 mm). This study emphasized the importance of the cut-type of onion strips. Furthermore, DIC treatment allowed a reduction on 99.9% of the initial total account of microorganisms in raw material.

On the other hand, Albitar et al. [17], instead of using DIC as a texturing treatment to improve onion drying, applied DIC as a blanching–steaming pre-treatment. Fresh white onions were cut (50 × 5 × 2 mm^3^) and then subjected to DIC treatment under 200 to 500 kPa for 5 to 15 s. Because, in this case, the target was to avoid texturing, the treatment was carried out far from the glass transition zone and without crossing the glass transition, and for that DIC was applied directly on fresh onions (4.50 g H_2_O/g db). CAD was carried after DIC treatment at 40 °C, 1 m s^−1^, and 267 Pa partially pressure humidity hot air until a final water content of 0.05 g H_2_O/g db. DIC treatment intensified the mass transfer by reducing the total drying time from 3200 min (untreated samples) to 700 min (DIC samples). Compared to the only CAD, DIC pre-treatment dramatically increased the effective diffusivity and the initial starting accessibility by 223% and 40%, respectively. Optimal DIC conditions were identified under 460 kPa and 14 s.

Téllez-Pérez et al. [55] evaluated the effect of swell-drying on green Moroccan pepper (*Capsicum annuum*) and studied DIC texturing under 0.20 g H_2_O/g db, between 100–600 kPa, and 5–35 s. Results showed that the drying time reached a final water content of 0.05 g H_2_O/g db and was reduced by a factor of 1.7 times thanks to DIC. Moreover, under a DIC treatment of 600 kPa for 20 s, the effectiveness of diffusivity was 246%. Besides, DIC treatment improved the starting accessibility and the effective diffusivity of rehydration kinetics by 125% and 272%, respectively.

Yuca, also named cassava (*Manihot esculenta*), is consumed after dehydration as flour. However, in the case of the sun and hot air-drying, a significant deterioration of the quality has been mentioned. In this respect, Setyopratomo et al. [7] studied the effect of swell-drying on fresh cassava roots in drying kinetics, physical product properties, and microbial decontamination. DIC texturing was carried out on pre-dried cassava roots between 025 to 0.33 g H_2_O/g db, under 260–540 kPa, and 12–48 s. It was possible to increase the water-effective diffusivity up to 2.8 times thanks to DIC. Moreover, relative to CAD, the water holding capacity (WHC) and the oil holding capacity (OHC) increased to 6.6 and 5 times, respectively. According to the authors, these results could be linked to increasing the total pore volume and the specific surface of swell-dried products. According to the authors, these results could be linked to an increase in the total pore volume and the specific surface of swell-dried products. Finally, DIC treatment also performed an 85.7% reduction of the initial bacteria content, which means that under accurate thermo-mechanical treatment conditions, it could be possible to achieve complete decontamination.

**Figure 5:**
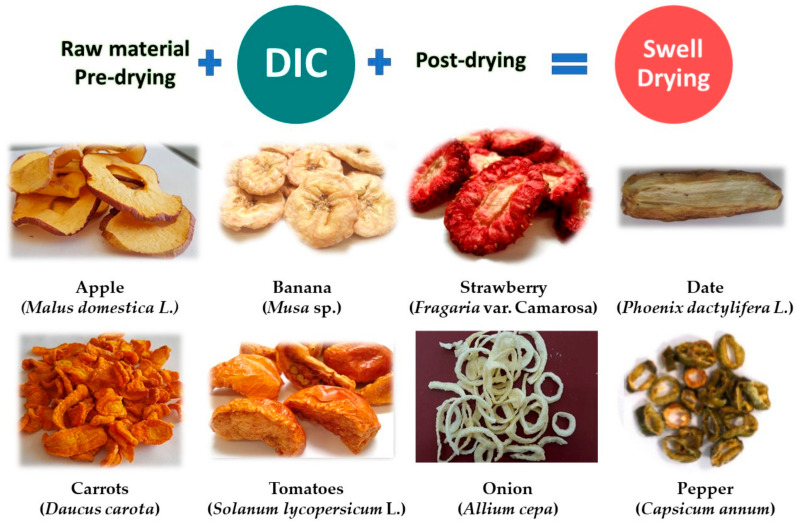
Examples of swell-dried fruit and vegetable products.

Many other fruit and vegetable products have been submitted to swell-drying, such as broccoli [13], okra pods [56], and beetroots [57], among others. In all the cases, it has been highlighted that DIC treatment easily intensifies the drying kinetics, reduces energy, and achieves the desired quality attributes of final products (e.g., preservation of bioactive molecules and microstructure expansion). Figure 5 shows some examples of swell-dried fruit and vegetable products.

On the other hand, concerning the paradoxical drying stage, Al Haddad et al. [58] evaluated the coupling of swell-drying to microwaves (MW) into apples and sweet potatoes. As previously indicated, DIC texturing improves the diffusivity of water under liquid and vapor forms. Then it could remedy the highest resistance phenomenon of CAD. However, microwaves assisted by air were applied as a second intensification method to fix the paradox stage. Microwave drying after the swell-drying treatment made it possible to reduce the drying time from two hours for DIC couple to CAD to less than 5 min for DIC couple to MW; water content was reduced from 0.20 to 0.05 g H_2_O/g db. Because at the final drying stage, the central part for removing water is governed by the gradient of internal vapor partial pressure. The use of microwaves leads to deep heats between the core and the surface area of the product, thus improving the drying kinetics.

### 3.2. Impact of Instant Controlled Pressure-Drop Treatment on Cereals Drying

Cereals represent an essential source of carbohydrates, proteins, and other nutrients necessary for human feeding. According to the Food and Agricultural Organization (FAO), cereals stand out as the main food grown worldwide. Maize, wheat, and rice are the most consumed products [59]. In this respect, DIC treatment has been studied on various cereals to evaluate its effect on grain quality preservation and drying processing.

All cereals are starchy foods. Starch has great nutritional, pharmaceutical, and industrial significance due to its unique physical, chemical, functional, and nutritional properties. A starch modification is any change in the structure of the starch molecule caused by various environmental, operational, and processing factors. These modifications may exert either positive or negative effects on the structure and functionality of starch molecules. Thermo-mechanical processes can induce several changes in starch, such as the variation in particle size, surface properties, solubility index, water absorption, swelling capacity, pasting, and gelation ability of starch [60,61]. Table 4 summarizes some examples of DIC treatment of cereals.

Maize, *Zea mays*, is one of the most widespread grains; e.g., in 2018 alone, a total of 1147 million tons were produced worldwide [59]. Zarguili et al. [62] tested the gelatinization properties of native starch when treated with DIC by applying different pressures and times to a 0.5 cm high amount of native starch. Treatments from 100 kPa to 500 kPa were performed for 2 min to test the effect of pressure, and at 300 kPa for 30 s to 900 s to measure the impact of time. The pressure value impacted the increase in gelatinization degree from 23.4% to 100%, and was correlated with a saturated steam pressure at 200 and 500 kPa, respectively. Furthermore, the starch system reached complete gelatinization. In native maize starch, gelatinization enthalpy was 11.6 J/g. It steeply decreased to 0.57 J/g after 15 min processing time, along with a rapid gelatinization observed during the first minute of treatment.

On the other hand, Maache-Rezzoug et al. [63] found that for standard maize starch, waxy maize starch, potato starch, and wheat starch, the enthalpy value was lowered when the treatment harshened. For treated cereal starches, there was a positive correlation of pressure and time to the transition temperatures. Moreover, a narrowing of the width of gelatinization endotherms into waxy maize starch was noted, increasing the enzymatic hydrolysis sensibility [63,65].

Maache-Rezzoug et al. [65] also evaluated the DIC effect on pasting properties of standard maize waxy maize, potato, and wheat starches. Their results showed an increase in pasting due to the pressure effect, influencing the viscosity and pasting time. Yet, time reached a peak to further decrease viscosity, as part of the explanation is that the integrity of starch granules was preserved for all the conditions. Still, their crystalline organization changed, affecting their functional properties [63,65], yet for potato starch, the viscosity dramatically increased in all treatments [63]. As part of microstructure changes, standard maize starch and wheat starch complexes of amylose–lipid was formed at the treatments of 200 kPa for 60 min and 300 kPa for 30 s, respectively, without leaching amylose [63].

Zarguili et al. [66] took standard maize starch and measured the water activity coefficient (γ) and mass transfer coefficients, which both reduced with pressure going from 100 kPa to 300 kPa. The water coefficient (γ), had a 43% reduction, from 5.86 to 3.36, with a steep decline from 100 kPa to 200 kPa (5.86 to 3.71). The mass transfer coefficient decreased, seeing a steep drop from 100 kPa to 200 kPa, as the values were 5.89 × 10^−5^ m/s, going through 0.92 × 10^−5^ m/s and finally 0.77 × 10^−5^ m/s at 300 kPa; this transfer coefficient was also affected by the gelatinization taking place, therefore, adding complexity to the model. On the other hand, Bahrani et al. [67] compared DIC to direct vapor and reduced pressure heat treatments on standard maize starch. This study found that values of perceived particle sizes increased as swelling capacity increased and agglomerates were formed, thanks to gelatinized granules. For all treatments, as the intensity increased, there was a reduction of the consistency coefficient and yield stress of starch suspensions with increasing process intensity. At 300 kPa, there was no difference between the treatments, as complete fluidization of starch suspensions was observed, and the elastic modulus (G′) decreased dramatically (G′ < 1 Pa), therefore the loss of rigidity and disappearance of granular integrity was apparent.

Paddy rice (*Oryza sativa*) must undergo a drying operation to avoid microbiological and physical damage. In this respect, despite the diversity of drying technologies, physical damage has been consistently reported, leading to a significant rate of broken grains. In addition, the traditional drying of paddy rice is a long process (18–24 h). In contrast, by applying, as a post-harvest treatment, DIC technology under saturated steam pressure of 400–500 kPa for less than 30 s, some gelatinization of the starch was observed, with a total hot air-drying time of about 2–3 h and a considerable reduction in the breakage rate (2–3% compared to 20–30%, by conventional operation).

Moreover, the whole white grain yield becomes 68–70% after polishing, indicating that DIC treatment remarkably preserves rice grains against ruptures and damage [68]. Furthermore, DIC also improved the tenderness of rice. It usually takes around 18 min for rice to absorb water and become tender, but thanks to a DIC treatment (500 kPa for 16–30 s), rice grains were cooked entirely for six min, a third of the time needed to cook untreated rice. Also, DIC preserved aromatic rice properties longer and widened the interval before the rice would be considered overcooked, from 18 min vs. 20 min. Then, DIC technology changed the drying kinetics of rice and its sensorial profile [3,69]. Moreover, the DIC treatment completely eliminates weevils, and the paddy rice can then be preserved for several years.

Expansion of biological matrices is of fundamental importance in snack products. In this respect, Yağcı [70] evaluated the effect of DIC texturing on some physicochemical and nutritional properties of puffed wheat snacks products (*Triticum* spp.). Snacks were produced by submitting moistened wheat under DIC texturing (300–500 kPa and 3–11 s) followed by drying in a hot air dryer. The best expansion and sensorial characteristics were obtained under a pressure of 500 kPa for 7 min, corresponding to the highest evaluated processing pressure at a low water content. Besides, it was highlighted that it could be possible to improve expansion and textural properties by increasing processing pressure and time. Still, it leads to the darkening of the raw wheat color.

### 3.3. Impact of Instant Controlled Drop Treatment on Animal Origin Food Drying

DIC texturing has also been applied to animal products such as milk, cheese, eggs, chicken meat, and seafood. Evaluation of the physicochemical properties and safety of products allows the optimization of DIC parameters.

#### 3.3.1. Dairy Food

One of the first studies on animal origin was done on skim milk powder (Table 5). As the drying rate becomes reduced due to case hardening and particle size, Mounir et al. [71] evaluated the impact of two kinds of DIC treatments to improve some physicochemical characteristics of milk powder. The first treatment was called “High Air Pressure Instant Controlled Pressure-drop”, which instead of using steam to increase pressure, uses compressed air ranging from 200–800 kPa. The second treatment used saturated steam, with pressures ranging from 100–700 kPa. In both processes, the level of pressure was the most influential factor in the final bulk density. Moreover, both processes reduced the amount of fine powder and increased the particle mean size (200 µm, compared to 100 µm for untreated spray-dried powder). In general, these results showed an improvement in the functionality of milk powder in terms of wettability, solubility, and dispersibility.

On the other hand, as the prevalence of allergy to cow′s milk proteins was increased, Boughellout et al. [78] also studied the impact of DIC treatment on milk protein immunoreactivity. Both bovine caseins and whey proteins were studied. The first one received a treatment of 400 kPa for 25 s and the second one received two different treatments, 400 kPa for 25 s and 600 kPa for 25 s. The results showed different behaviors when tested with gel electrophoresis (SDS-PAGE). Bovine casein proteins increased their IgE binding, increasing its allergenicity, and whey proteins reduced their IgE binding; these results were confirmed through Indirect ELISA and Western Blot. It is worth noticing that the average response of whey proteins treated at 400 kPa is reduced by half, while those treated with 600 kPa were 32%, which could be linked to a change in the molecular structure of the proteins.

Besides milk, Mounir et al. [72] evaluated the impact of DIC treatment on the feasibility of developing new cheese snacks. For that, pre-dried Leerdammer cheese was submitted to DIC treatment under a surface response methodology design, with pressure ranging from 200–550 kPa for 10 to 30 s. The best sensory characteristics, which means crispy cheese cube snacks and expanded granule cheese powders, were obtained under a DIC treatment of 550 kPa for 30 s. This study showed that DIC cube cheese snacks expanded up to 36 times their original size. DIC powder cheese granules presented an improved porosity by exhibiting a lower bulk density value, with particle sizes of around 250 μm, and higher compressibility values. Researchers also observed a high dependence on steam pressure, which means that the process can be easily tweaked and adjusted according to the consumer′s needs. On the other hand, El Zahar et al. [73] obtained expanded granule cheese powders with a low bulk density and a high specific surface area, which means a better functional quality as a food ingredient.

#### 3.3.2. Eggs Products

When comparing the effects of DIC treatment on egg drying (both yolk and egg white) to conventional airflow drying (CAD) and freeze-drying (FD), Mounir et al. [74] showed that DIC powders performed high functional quality attributes (Table 5). Then, concerning egg powders′ bulk density, porosity, and oil binding ability, both DIC and FD treatments showed the best results regarding CAD. On the other hand, DIC performed the best water absorption capacity, emulsifying capacity, foaming capacity, and stability. Moreover, it was found that the pressure parameter was the most influential factor in egg white for porosity, water absorption capacity, emulsifying capacity, foaming capacity sharing said prominence with egg yolk in all except the water absorption capacity, oil binding ability, and emulsifying capacity, having a certain effect in the last two which is to be expected.

#### 3.3.3. Poultry Products

Mounir et al. [18] and Mounir [75] reported using Instant controlled pressure-drop as a novel pre-treatment of fresh chicken breast to develop a newly expanded texture on dried meat (Table 5). First, the fresh chicken was submitted to a DIC treatment under 400–700 kPa and 82 to 130 s, and subsequently, it was dried under CAD at airflow speeds higher than the critical velocity. Results showed that DIC pre-treatment triggers an expansion ratio 15 times higher than untreated samples (CAD). Moreover, the highest relative surface expansion value was obtained at 530 kPa and 110 s, with 178% compared to the raw chicken breast. The main factor influencing the expansion ratio with a positive correlation was the saturated steam pressure. Besides, DIC texturing intensified the drying kinetics of chicken meat. And, by comparing the rehydration behaviors of DIC-treated samples to freeze-dried products, DIC improved the water retention properties of 3.50 g H_2_O/g db for the 700 kPa and 110 s treatment 3 g H_2_O/g db for freeze-dried meat. CAD chicken products exhibited the worst rehydration ability, which could be correlated to the shrinkage and thermal degradation exhibited due to the lengthy drying process, damaging the myofibrillar and collagenous connective tissue proteins. The treated products showed very high functional properties after grinding, thanks to its new expanded microstructure. Electron microscopy analysis of DIC treated samples exhibited a porous structure with an array of capillary vessels with randomly spaced holes of various shapes and sizes. The CAD had a compact structure of fiber rods, and the freeze-dried samples showed broken cell walls with straw-like geometries derived from the freezing process. In this respect, it is also important to highlight that DIC is a flexible process, and it can be optimized to meet different criteria of product quality.

#### 3.3.4. Seafood

Haddad et al. [76] studied the effect of two technologies, swell-drying (DIC + convective hot air drying) and Multi-Flash Autovaporization (MFA) or Dehydration by Successive Pressure-drops (DDS), in thawed salmon (*Salmo salar*) and tuna filets (*Thunnus albacore*) (Table 5). Strictly speaking, DDS was defined as a drying operation based on multiple cycles of instantaneous decompression towards the vacuum until the required dehydration percentage is achieved. Moreover, this technology employs air as a compressed fluid instead of using steam as DIC to increase pressure. In the case of DIC texturing, fish cubes with initial water content between 0.43 to 1.56 g H_2_O/g db were submitted under pressures ranging from 340–660 kPa and time ranged from 7 to 23 s. For DDS, fish cubes with the same initial water content were submitted under pressure between 260 and 540 kPa for 4 to 46 s. The results showed that DDS provided the shortest overall drying time, linked to an almost complete absence of shrinkage, therefore maintaining high levels of diffusivity comparable to swell-dried samples. DDS provided no significant effect on density for both samples. On the other hand, DIC provided a better rehydration behavior of products, this thanks to a more porous structure. Besides, regarding the sample density, the rehydration capacity, the water activity, and the color, this study showed that both technologies (DIC and DDS) demonstrated better quality and functionality than only CAD.

Shrimp meat (*Penaeus notialis*) has also been submitted to DIC by coupling it with intermittent microwave MW/airflow drying (IMAD) [77]. Before any DIC treatment, fresh shrimps were blanched (5 min in boiling water), peeled, and cut into cubes. After that, they were submitted to DIC texturing under a steam pressure range of 400–700 kPa and treatment time of 70 to 130 s. Subsequently, they were treated with various IMAD cycles that submitted DIC textured shrimps to convective microwave power during 30 s, followed by 60 s of airflow (2.5 ms^−1^) at ambient temperature (23 °C) till the end of drying. The evaluated MW density power was 6, 12, and 24 W/g wb (wet basis). A domestic microwave oven with a frequency of 2450 MHz and a maximum power output of 900 W was used. Results showed that the drying kinetics improved by coupling DIC (700 kPa and 130 s) with IMAD. To reach a final water content of 0.05 g H_2_O/g db, DIC + IMAD required only 2 min, instead of 30 min by IMAD. Moreover, regarding consumer acceptance, positive results were obtained after hedonic trials of odor and crispiness. Thus, by coupling DIC to IMAD, it could be possible to develop high-quality shrimp snacks.

As it has been shown through numerous studies, the Instant controlled pressure-drop technology has proven to be a handy tool in food drying. It enhances the traditional drying methods by reducing the total drying time, texturing food material, and improving the final products′ nutritional and functional properties. Moreover, DIC technology can be coupled to other emerging drying techniques such as microwaves. Besides, swell-drying perfectly farmers′ needs for decentralized processing solutions in fruit and vegetables. As pre-drying can be done in the production zones by conventional drying (e.g., hot air drying, solar or sun drying), and DIC treatment can be carried out in a cooperative center; fresh products can easily be transformed into high-quality dried end-products. Finally, DIC provides new opportunities to develop new food products, such as snacks free of additives and new ingredients for baby food, instant soups, and bakery.

## 4. Instant Controlled Pressure-Drop Process on Food Components Extraction

The growing importance of the extraction of plant-based active molecules and, more generally, from natural raw materials at the expense of synthetic general chemical molecules marks the pharmaceutical and food industry strategically [79]. The health effects of synthetic molecules often seem too limited, sometimes leading to problems of resistance generation in treated subjects. On the other hand, complementary molecules, even in traces, from a plant often show synergistic effects in the proposed treatments [80]. Conversely, the extraction of the desired molecules from plants is often characterized by low yields and too long extraction kinetics. Then, new technologies capable of enhancing extraction are being applied. An adequate intensification of these extraction operations can lead to a broad extension of these natural active molecules′ manufacturing, marketing, and use. Such intensification would allow the proper valorization of plants already identified for hundreds of years for their health benefits in various cultures. In this respect, the DIC technology has proved to be a key tool to intensify the extraction of natural active molecules of food and plant materials by increasing yields, improving the extract quality, and being a green and sustainable process. DIC as a pre-treatment/texturing process greatly intensifies the solid-fluid interactions through a higher porosity of the solid matrix. Indeed, such texturing allows the product to remove the internal water and increase the solvent transfer. These changes result in an increase in the diffusivity of water and solvents within the solid matrix [81]. The following section describes some representative examples of the effect of DIC treatment on food and plant biomolecules extraction (Table 6).

### 4.1. Essential Oil Extraction

The industrial extraction of essential oils (EOs) is widely artisanal and uses traditional extraction methods such as hydrodistillation and solvent extraction, which has changed little over the years. These processes are characterized by high energy consumption and by lead to undesirable changes of natural compounds, such as rearrangements, isomerization, oxidation, and racemization reactions [84,85]. Moreover, in solvent extraction, dangerous solvents could remain as traces, which lowers the final quality of EOs [88]. For that reason, innovative processing techniques for biomolecules extraction have been developed, such as microwave-assisted extraction and supercritical fluid extraction. However, they are only well-controlled at a laboratory scale. Thus, they are unable to fulfill industrial requirements [84]. In this respect, DIC technology has performed outstanding results to enhance the extraction of essential oils of bioproducts at laboratory and industrial scales.

*Hyssopus officinalis* is a fragrant flower whose essential oil is used as a flavoring agent in prepared food, meat, and candied products. The main components of hyssop essential oils are inocamphone and isopinocamphone, two bicyclic monoterpene ketones [82]. Challenged against hydrodistillation (HD), Ultrasound-Assisted Extraction (UAE), and Soxhlet Extraction (SOX), extraction of essential oils by DIC proved to be the best way that leads faster to higher yields and better quality EO. Generated essential oil had a higher concentration of oxygenated components and less non-oxygenated sesquiterpenes. The optimized factors for essential oil yield were 100 kPa and 12 cycles of 100 s each. The fact that the number of cycles was the most influential factor can be explained mainly as an improvement consequence of the autovaporization process. The alveolation effect observed in micrography would also take part in such an intensification process [82].

On the other hand, Feyzi et al. [83] also compared the effect of DIC against hydro-distillation, ultrasound-assisted extraction (UAE), and soxhlet extraction of *Bunium persicum* essential oils. Their results proved the efficient effect of DIC on the quality and quantity of the obtained essential oils. By comparing DIC vs. UAE, an important advantage of DIC was its selectivity to extract oxygenated components. This difference could be linked to UAE cavitation, which improved the extraction of non-oxygenated components due to solvents. Moreover, DIC performed a better quality of essential oil than Soxhlet essential oils, which presented high undesirable non-polar components and high chain hydrocarbons; likewise, it required high usage of organic solvents.

Common myrtle (*Myrtus communis* L.) is a fragrant leaf. The essential oil extraction of this plant was performed in two ways, first directly by the DIC equipment and then by a final extraction of the solids. Hydrodistillation (HD) was used as a control. Results showed that hydrodistillation with untreated myrtle leaves had a yield of 0.51 ± 0.04 g EO/100 g db, and the optimized extraction by DIC (600 kPa, four cycles of 30 s) obtained a yield of 0.56 ± 0.12 g EO/100 g db. It is worth noting that this roughly 10% yield improvement could be achieved with only 2 min of DIC treatment instead of 180 min for HD. Additionally, the obtained EO by DIC produced a better quality; this was revealed by a strongly spiced and odoriferous fragrance and an improved antioxidant activity [85].

Lavender Grosso (*Lavandula intermedia* var. Grosso) is a sterile clone issued from hybridization between *Lavandula angustifolia* Miller and *Lavandula latifolia* Vill [89]. This plant has the characteristics of high essential oil yields, a very robust culture, and the presence of camphor [6]. On the other hand, it has been highlighted that the composition and yields of the generated EO can vary depending on environmental factors and the extraction method [90]. Its EOs are very commonly used in the cosmetic, pharmaceutical, and household cleaning industries. Its effects have been widely researched, and include its insecticide activity and its anti-inflammatory, sedative, and anti-bacterial attributes [91]. The most common method for EO extraction from lavender is steam distillation, but solvent extraction is also widely used. By looking for new EO extraction alternatives, Besombes et al. [6] studied the feasibility of DIC technology at laboratory and pilot plant scales to extract Lavender EOs. To evaluate the DIC performance, this was compared to the hydrodistillation process (HD). In both cases, the generated EOs were analyzed through GC–MS, and the residue material was analyzed by solid-phase microextraction. Results showed that DIC enhanced the EO yield by 4.25 g EO/100 g against 2.3 g EO/100 g for HD.

Moreover, the extraction time was reduced from 2 h (HD) to only eight min for the DIC process. On the other hand, scanning electron microscope images of lavender before and after DIC treatment showed that raw material had a compact and relatively homogeneous internal structure, while DIC-treated plants presented a porous texture. Finally, DIC treatment required less energy and water than steam distillation, consisting of 662 kWh and 42 kg water/t of raw material.

Rosemary leaves, *Rosemarinus officinalis* L. are increasing their popularity in the food industry due to their antioxidant capacity. Rosemary leaves are adapted to drought for having a thick cuticle and a dense abaxial indumentum [10]. The oils impart fragrance, and flavors should be removed from the leaves to obtain a pure antioxidant extract. Allaf et al. [10] found that by DIC extraction 6.25% more essential oil per 100 g of dry material was obtained, the amounts being 1.28 ± 0.04 g EO/100 g db for HD, vs. 1.36 ± 0.08 g EO/100 g db for DIC treated leaves. Moreover, DIC essential oils improved quality due to an 8.44% higher content of oxygenated compounds, which have much more value for the flavor and fragrance industry.

In another study conducted in orange peels by Allaf et al. [14], *Citrus sinensis* was treated by DIC to extract essential oils directly. The applied treatment was 600 kPa and 11 cycles with a total treatment time of 152 s. DIC extraction yielded 1.66 ± 0.045 g/100 g db, representing a 98.97% extraction efficiency. By comparing these results to HD, DIC represents an increase of 4.2% of the extraction efficiency in only 2.5 min vs. 4 h [14].

DIC EO extraction sorts out the hurdle of the compact and relatively homogeneous internal structure of raw materials. In fact, by changing the microstructure of plant and food materials, the release of the essential oils from the glands can be increased.

### 4.2. Antioxidants Compounds Extraction

Antioxidants in plants belong to a group known as secondary metabolites. Chemically, antioxidants can be phenolic compounds and vitamins, among others [92]. On the other hand, food drying through thermal processes tends to decrease antioxidant concentration. Then, new drying processes must be applied to preserve the beneficial properties of antioxidants. In this respect, DIC could be an excellent way to dry food while preserving antioxidants. This section contains several studies about the application of DIC to extract bioactive molecules, and Table 7 summarizes the most important findings.

The industry of citrus juice extraction generates peels and pulp, which still have compounds of interest, such as phenolics [14]. Before DIC treatment, industrial orange by-products had a compact and relatively well-organized cell structure. DIC treatment led to a more porous structure. In this respect, an increase in the extraction efficiency of phenolic compounds through expansion of the structure was observed in orange peels after DIC treatment [43]. The extraction of flavonoids was optimized at 490 kPa for five cycles and a total heating time of 186 s. DIC increased the extraction of hesperidin by 537%, rutin by 236%, flavone by 117%, and naringin by 160%.

On the other hand, Ben Amor et al. [93] studied the effect of DIC treatment in the extraction kinetics of anthocyanins from the Malaysian Roselle, *Hibiscus sabdariffa*, or “flor de Jamaica”, expressed as Total Monomeric Anthocyanins. This study found that thanks to the increased alveolation of the tissues triggered by DIC treatment, the effective diffusivity of anthocyanins increased from 4.19 × 10^−11^ m^2^/s for untreated raw material up to 6.11 × 10^−11^ m^2^/s. Moreover, after optimizing the treatment (180 kPa and one cycle of 18 s), the anthocyanin extraction yield improved by 135%. Besides, DIC extracts had no variation of the anthocyanin fractions of Delphinidin 3-sambubioside and Cyanidin-3-sambubioside, representing 85% of the anthocyanin profile of Roselle. The DIC process increased the transfer of antioxidants to solvent through cell structure modification.

Pomegranate peel wastes are increasing since pomegranate juices consumption has also increased. Being a colored waste, it is an interesting matrix for antioxidant extraction [94]. With the optimized setting of 300 kPa and 1 cycle for 35 s, an 8.25% improvement in total phenolic content was obtained compared to raw pomegranate peel, from 38.77 to 46.02 mg GAE/g db. It has been highlighted that in the beginning, various DIC extraction cycles were studied (from 1 to 5); however, to avoid the risk of losing valuable phenolic compounds due to autovaporization, the number of DIC cycles was optimized to one. On the other hand, regarding the radical scavenging of extracts, DIC extracts provided 74.12% of inhibition against 62.10% for raw material [94].

Olive leaves, *Olea europaea* L., also carry important fragrant molecules and antioxidants; among the latter categories, the seven defining Olive leaf polyphenols are oleuropein, apigenin-7-glucoside, hydroxytyrosol, luteolin-7-glucoside, tyrosol, vanillic acid, and verbascoside [95]. Mkaouar et al. [95] evaluated the total phenolic compound yield after DIC drying and texturizing olive leaves. The yield was higher than that of untreated material with an optimized value of total phenolics of 248.6 mg Gallic Acid Equivalent/ g at a saturated steam pressure of 100 kPa for 11 s. Mkaouar et al. [96] obtained a 176% improvement in total phenolic content extraction over the raw material after DIC treatment. Moreover, DIC extracts demonstrated a better antioxidant capacity [97]. From the three studies by Mkaouar et al. [95,96,97], DIC treatment caused a microstructural modification of olive cells by creating alveolation. This results in better conditions for solvents to reach the compounds of interest seen from the scanning electron microscope.

Chokecherry (*Prunus virginiana* L.) is rich in bioactive molecules such as phenolics, which have antioxidant properties. A DIC treatment of 270 kPa for 20 s preserved the content of total phenolic compounds, flavonoids, and, more importantly, kuromanin, the main anthocyanin present in chokecherry. Furthermore, the optimal DIC operating parameters preserves chokecherries′ antioxidant content and activity at ambient temperature [98].

Winemaking produces a great number of residues, such as grape seeds and grape stalks. These are considered at the same time as a rich source of phenolic compounds and pollutants [101]. Thus, to improve the extraction of phenolic compounds from grape stalks, Sánchez-Valdepeñas et al. [99] applied DIC as a pre-treatment to extract the phenolic compounds. During the extraction of phenolics, one of the most influential factors is the size and porosity of the solid particles to ensure solid solvent contact. Then, DIC treatment could provide higher porosity and improvement of the specific surface area, increase solvent diffusion inside of powder particle, and consequently increase the availability of desired compounds. This effect was achieved after treating grape stalks powder under 300 kPa, 50 s. The yield of individual phenolics was highly increased, especially for quercetin, gallic acid, and ellagic acid.

DIC technology was applied by Wang et al. [100] to obtain a suitable cell disruption in green tea processing. Cell disruption is fundamental in green tea processing since mechanical and thermal stress can result in unfavorable characteristics such as oxidation and poor visual quality [102,103]. In this respect, the DIC micro-structured matrix increased cell disruption in a precise manner. The treatment resulted in distorted cells, widened space between cells, and disrupted and rearranged cellular membrane in tea leaves. In addition, the DIC sample showed 2.5 times increased redness over the control, which was spread naturally for 24 h. It is also worth noting that the DIC treated tea showed a better infusion behavior of tea polyphenols and amino acids. The enhancement in tea polyphenols content for the first brew from twisted and needle tea was about 35% and that from flat tea was about 15% in the DIC method over the traditional processing.

### 4.3. Vegetal Oil Extraction

As happens with the extraction of other biomolecules, vegetal oil extraction takes two main stages; first, the superficial oil quickly migrates to solvent, but in the second stage, the oil must migrate more considerable distances until it reaches the surface [104]. The search for technologies that modify the raw material structure to favor the migration of molecules has increased in the last decades. On this basis, DIC technology has emerged as an excellent option to modify cell structure and improve the extraction of oily materials, as shown in the studies listed in Table 8.

Allaf et al. [5] applied DIC on the rapeseeds seeking to improve oil extraction. Under the best treatment conditions (510 kPa, 50 s), the oil yield was increased from 0.25 to 0.36 g oil/g db. It was supposed that DIC treatment modifies the rapeseed structure, favoring the migration of triglycerides to solvent extraction.

Bouallegue et al. [105] applied DIC treatment in several oilseeds, using higher pressure (630 kPa) and different times depending on the kind of oilseed. For all seeds tested, an average yield growth of 10% was obtained. The increase in yield was attributed to the higher availability and better kinetics of solvent extraction triggered by DIC. Moreover, higher extraction yields were coupled with consequent preservation of the product quality. Another advantage of applying DIC as a textural pre-treatment to oilseeds is reducing extraction time [110].

Soybean oil (*Glycine max*) is one of the most widely consumed cooking oils. The cracked seeds are set in touch with solvent to obtain soybean oil. However, like other seeds, this process is time-consuming, and the heat pre-treatment applied to the seeds can cause degradation of the oil. Jablaoui et al. [106] used DIC to texturize soybean seeds as pre-treatment to increase oil yield. And DIC triggered an improvement in the extraction yields and kinetic extraction, which was essentially generated by a change in the technological aptitude of the treated grains. Furthermore, with DIC requiring a short time process, oil quality was not affected [106,111].

Eikani et al. [107] worked on the bio-oil extraction of one edible oil industry staple, safflower oil, and a comparison was made for treated against untreated samples; for safflower oil, the yield increased by 70.43%, from a yield of 17.52% wt (the weight ratio of extracted oil per feed mass in a dry basis) to 29.86% wt.

Cold-press oil extraction usually yields an oil of high quality but low quantity, and the press-cake still contains around 15% oil. Zeaiter et al. [108] applied DIC as pre-treatment to modify the structure and texture of sunflower seeds. The yield in DIC treated seeds was improved by approximately 11%, and the content of specific fatty acids was increased; for instance, linoleic and oleic acids yielded 46% and 33% more, respectively. The best conditions of pressure and time to assure the highest yield was 500 kPa and 50 s. DIC treatment possibly increases the oil availability resulting from the higher porosity and the broken cell walls by DIC-texturing triggered on the matrix.

It is known that the plant′s natural tissue structure resists solvent diffusion, which slows down the extraction process, which results in larger amounts of solvents needed and higher energy and time consumption. Due to these drawbacks, DIC technology has been widely explored as an alternative for extracting many compounds of interest such as essential oils, antioxidants, vegetal oil, and even making it suitable for the remotion of potentially harmful and toxic substances from the raw material. One of the things that these metabolites have in common is their thermolability and fragility against high temperatures. Since the commonly used extraction methods usually involve prolonged exposure to harsh solvents, the sought-after characteristics of the extract are often affected. This is not a concern in a DIC operation since there are no solvents involved, and the time of exposure is negligible in comparison. In other words, DIC lowers the processing time, preserving the desired features.

## 5. Instant Controlled Pressure-Drop Process on Food Safety

According to the World Health Organization, the consumption of food contaminated by bacteria, viruses, parasites, or chemical substances such as mycotoxins or heavy metals, among others, causes more than 200 diseases [112]. On the other hand, food allergies are also an important public health problem, with eggs, milk, peanuts, tree nuts, soy, wheat, shellfish, and fish being the most common food allergens [113]. Therefore, food safety is an essential tool in the fight against foodborne diseases. Food safety faces four major challenges: (1) microbial safety, (2) chemical safety, (3) personal hygiene, and (4) environmental hygiene [114]. In this regard, DIC processing has had a positive impact on microbial and chemical safety. Various studies have shown that DIC′s thermal and mechanical mechanisms can effectively inactivate vegetative and spore-forming bacteria [7,8].

Moreover, thermomechanical treatment has also reduced allergens in food products such as lupins, pistachios, and nuts [115,116]. Additionally, our research group is presently studying the effect of DIC in mycotoxin decontamination. The following section describes the most relevant results about the impact of DIC technology on microbial and mycotoxin decontamination and allergen reduction.

### 5.1. Microbial Decontamination

Foodborne diseases are caused by food contamination and occur at any stage of the food production, delivery, and consumption chain. The most prevalent diseases are caused by microorganisms such as *Listeria monocytogenes* and *Staphylococcus aureus* [117,118]. The symptoms related to these diseases are broad, from diarrhea to cancer [112]. It is thus imperative to find new methods to decontaminate food to avoid or at least decrease the incidence of foodborne diseases.

The total count plate method evaluated the decontamination effect of DIC (400 kPa for 12 s) on Cassava flour (*Manihot esculenta*). DIC treatment results in greatly decreasing the initial bacterial content of raw material decreased to 85.7%, from 605,000 CFU/mL for fresh cassava to 86,500 CFU/mL for DIC samples [7]. Swell-drying onions also performed significant decontamination. In fact, under a DIC treatment of 350 kPa and 15 s, the initial total count plate of fresh onions (875,000 germs/g) was reduced up to a ratio log F of 3.9 (100 germs/g) [17].

On the other hand, regarding the effect of DIC treatment on spores, the study of Debs-Louka [8] showed that DIC triggered the spore destruction of *Bacillus stearothermophilus* at 280 kPa for 30 s. Moreover, it was identified that the higher generated steam amount within the cell, and the shorter the pressure-drop time, the more efficient the decontamination. In addition, in the function of food material, the decontamination can be improved by multiple cycles of DIC treatment [3].

### 5.2. Allergens Reduction

Food allergens are a health issue while consuming legumes and cereals. Legumes are a rich source of proteins; however, they contain IgE binding-proteins, which are responsible for the immunogenic response in the consumers. In decreasing order, legumes associated with allergenic reactions are peanut, soybean, lentil, chickpea, pea, mung bean, and red gram [119]. Therefore, it is imperative to remove allergens from food.

Beans, chickpeas, peanuts, lentils, and soybeans are significant sources of macronutrients, especially proteins, and as such, they are also a source of food allergens. Then, to take the maximum advantage of these products, Cuadrado et al. [11] studied the effect of DIC on their proteins, finding a reduction in the immunoreactivity correlated to a higher pressure and heat treatment time, 600 kPa for 180 s being the best treatment (Table 9). The total protein content for any seeds was not affected. Still, DIC reduced the relative band density for all bands in the immunoassay, except in roasted peanuts for just one band at 37 kDa showed an increase, and soybean bands were almost negligible.

Guillamón et al. [115] tested allergenicity for lupin seeds, yet with a different approach as the process was done through a western blot instead of an immunoassay. It resulted in a similar finding to Cuadrado et al. [11], as the harsher the DIC treatment, the better the results are, in this case, at eliminating the overall in-vitro allergenicity.

Moreover, DIC technology has been tested to counteract the allergenic potential of tree nuts, such as pistachio and cashew, which contain allergenic proteins. The influence of DIC technology was proven by Vicente et al. [116]. They evaluated the change in the allergenic proteins via western blot, using IgG anti-11S and anti-2S and IgE antibodies from sera of patients sensitized to pistachio and cashew. The DIC processing caused changes in the electrophoretic pattern, reducing the number and intensity of protein bands as the pressure and temperature treatment incremented, which resulted in the decrease in detection of allergenic proteins. After DIC treatment at 700 kPa and 120 s, the highest reduction in IgE binding was reached for pistachio (75%) and cashew (67.2%).

Not only legumes have developed allergenic responses, but also cereals. The most common cereal allergenicity is related to gluten [122]. Gluten is a protein in wheat, barley, and rye that is responsible for dough formation. However, many people experience digestive and health problems caused by eating gluten or wheat [123]. For wheat flour, *Triticum aestivum*, Mahroug et al. [121] studied the possible effects that DIC might have on gluten, which among those resulted in a sensory change in rigidity, as β-structures were enhanced. Indications of deep denaturation were also found; as pressure and time increased, α-helixes were lost, intermolecular β-sheet increased, and sulfide bonds were rearranged without changing the primary structure. The functional properties of wheat protein were affected, obtaining lower protein solubility, emulsifying capacity, and foaming ability at the highest DIC treatment conditions (700 kPa and 60 s). The changes in said properties suggest uses of wheat proteins other than only making bread. Regarding immunoreactivity to gluten, DIC-treated samples showed lower values than the untreated. However, more studies are needed to get a specific immunoreactivity, which is also patient-related.

## 6. Other Applications of the Instant Controlled Pressure-Drop Process on Food

### 6.1. Reduction of Anti-Nutritional Compounds

Legumes hold a special interest in the human diet as a vegetable source of proteins, yet most have an issue with adverse non-nutritional factors. Among these compounds are oligosaccharides, inositol phosphates, trypsin inhibitors, and lectins, which in high quantity could have adverse effects on health consumers, but in low quantity, they are beneficial. The first study applying DIC to reduce non-nutritional compounds was done by Pedrosa et al. [124]. These authors treated soybean, lupin, lentil, chickpea, and roasted peanut at 600 kPa for 1 min, finding a significant reduction in all the anti-nutritional compounds evaluated (Table 10).

Sarphonka seeds, *Tephrosia purpurea*, is a legume studied by Ben Amor et al. [125], who focused on improving oligosaccharides (ciceritol and stachyose) extraction. Results showed that for ciceritol, the pressure steam had a positive effect on its extraction, and for stachyose, the initial water content and the interaction between water content and steam pressure had a significant and positive impact. DIC treated seeds yielded 150% more ciceritol and 173% more stachyose. The optimal DIC conditions for both molecule extractions were defined as being 520 kPa for 197 s, and a water content of 0.15 g H_2_O/g db. Besides, regarding the optimal conditions per each compound, for ciceritol it was found to be 600 kPa, 240 s for 1 cycle, and for stachyose, 200 kPa, 30 s, for 1 cycle. The DIC operation decreased the time needed and reduced it by 75% to 1 h instead of 4 h for an untextured sample.

Rapeseeds are cultivated worldwide due to their high oil and protein content as well as their balance in essential amino acids [126]. This makes rapeseed a good candidate for use in human and animal nutrition. Nonetheless, its usage has been restricted by antinutritional factors and toxic substances such as glucosinolates and phytates. Various techniques have been developed to remove or decrease the number of glucosinolates and their by-products. Still, none of these techniques have been adequate since they can induce protein loss, incomplete inactivation, and on many occasions, they are time and money-consuming. Because of this, the DIC treatment was evaluated by Haddad & Allaf [126] on rapeseed meals. This study confirmed the efficiency of DIC as a suitable process for rapeseed detoxification. Glucosinolate content decreased by 40% after 1 min and 98% after 7 min of DIC treatment.

The common bean (*Phaseolus vulgaris* L.) is highly valued for human consumption due to its high nutritional value. However, it contains some secondary metabolites referred to as non-nutritional compounds. These compounds are synthesized and accumulated during the maturation of the seed for the germination process and, when ingested, can reduce the availability of nutrients, causing and flatulence and generating neurotoxic effects when consumed in high amounts; however, at low concentrations, they show a beneficial effect. Cardador-Martínez et al. [12] evaluated the change in the non-nutritional composition of bean seeds and sprouts treated by DIC. The results revealed that with germination, the concentration of phenolic and tannin compounds increased by 99% and 73%, respectively, and the concentration of saponins (65.7%), while phytates and trypsin inhibitors decreased 26% and 42%, respectively. On the other hand, DIC treatment leads to a phytate content of (23–29%), saponins (44%), and oligosaccharides increased in bean sprouts. It also decreased phenolic compounds (4–14%), tannins (23% to 72%), and trypsin inhibitors (95.5%).

### 6.2. Oil Deodorization

Deodorization is a steam stripping process wherein good-quality steam generated from degassed and properly treated feedwater is injected into soybean oil under low absolute pressure and sufficiently high temperature (210–270 °C) to vaporize the free fatty acid and odoriferous compounds and carry the volatiles away from the feedstock.

The role of deodorization in the processing and refining of edible fats and oils has long been accepted as the last step in preparing the oil for use as an ingredient in margarine, shortening, salad oil, cooking oil, hard butter for the confectionery industry, and many other products in the food industry [127,128]. Although oil deodorization is necessary to achieve a high final quality product, there are some disadvantages; one of these, for instance, is the formation of trans fatty acids [127,129].

Considering that a vacuum step is required during the DIC process, after applying high temperature and pressure, it is possible to theorize that fatty acids and other compounds responsible for the oil odor could be removed. Additionally, using Multi-Flash instant autovaporization (MFA), it was possible to be refined perfectly, including deodorization and/or de-solvation, using several DIC cycles. This significantly reduces volatile molecules at a much lower level than the international standard levels.

## 7. Conclusions and Perspectives

Since its invention up to now, the DIC technology has performed outstanding results in food processing. DIC has shown its potential to remedy the mass transfer resistance linked to cell collapse in drying operations. Thanks to the newly generated porous structure, an increase in the starting and the effective diffusivity of food products were observed. This intensification directly impacts the drying operation, reducing the total drying time (in some cases to 1 h instead of 6 h) and has improved the integral quality of dried products. As a result, swell-drying has become an innovative and low-cost operation to obtain nutritious dried products. It is worth noting that even if most of the studies have been done by coupling DIC to CAD, additional studies must be carried to evaluate the coupling of other drying technologies as infrared and microwave drying. Besides, as farmers can quickly adopt swell-drying, this technology can help to reduce the food waste of fresh products.

On the other hand, swell-drying answers consumers′ demand for healthy snacks, sustainable practices, plant-based food, and new fruits and vegetables, and keeps them free from additives and chemical preservatives. Moreover, swell-dried food products can be easily used as ingredients in baby food, instant soups, beverages, 3D printing food, etc.

In extraction operations, DIC performs excellently as a pretreatment and as an extraction operation itself. First, in the case of DIC as a pretreatment, the studies concluded that, thanks to the new microstructure, the plant/food materials were better at realeasing the desired (e.g., antioxidants) or undesirable molecules (oligosaccharides). Then, according to the selected operative parameters, optimizing the DIC as a pretreatment of conventional (e.g., hydrodistillation) and emerging extraction technologies (e.g., ultrasounds and microwaves) has been possible. Alternatively, DIC as an extraction operation has shown that by applying multiple cycles of DIC, it was possible to achieve the same yields of traditional methods in only some minutes. This operation has been called “Tripolium” which implies (1) A DIC autovaporization-texturing process, (2) An internal under-vacuum invading solvent flow, and (3) an external under-steam pressure expulsing solvent flow. In this respect, further research must be carried out to better apply this technology at an industrial level.

At the same time, DIC has performed satisfactory results in terms of microbial safety. Thanks to the thermomechanical impact on microorganism cells, DIC can be considered as a decontamination process for solid and powder food. Therefore, further studies should be conducted to understand better the fundamentals of microbial decontamination carried out by DIC treatments.

On the other hand, concerning chemical safety, DIC has shown its potential to reduce food allergenicity in some foodstuffs such as nuts; however, more studies could be carried out on eggs and seafood. In addition, the mechanism of food mycotoxins decontamination through DIC could be evaluated.

In addition, DIC has been used to reduce non-nutritional compounds of legumes, which could directly impact the consumption of plant proteins as human food and feed. In this respect, new functional food products could be developed now from dried legumes or with extracted proteins by DIC.

Finally, DIC can also improve other food unit operations, such as oil deodorization, desolventation, dehydrofreezing, blanching, coffee drying, and roasting, among many others. Then, it could be said that DIC is a disruptive technology with a high potential to redefine several food processing methods.

## Figures and Tables

**Table 1:** Summary of the DIC conditions applied to treat fruits and some key findings.

Matrix	Objective	Applied Treatment: Pressure; Time	Optimal: Pressure; Time	Key Findings	References
Apples (*Malus domestica* L.)	Improve drying kinetics of apple granule powder with DIC	200–500 kPa, 1 cycle, for 5 to 55 s	450 kPa, 1 cycle, 12 s	55% reduction of drying time	[27]
	Coupling of Osmotic Pretreatment to DIC to inhibit apple cubes deformation	95 °C; atmospheric pressure for 10 min, further decompression, and Vacuum of 4–6 kPa for 1 h	-	DIC helped with perceived hardness compared to HA drying, but the solutions improved the crispiness factor	[23]
	Compare the effect of pectin modification with DIC for texture analysis	95 °C atmospheric pressure for 10 min, further decompression and Vacuum of 4–6 kPa for 2 h	-	Pectin modification increased crispiness as comparable to MD and MCC Osmotic pre-treatment	[24]
	Explain the effect of water equilibrium process on expansion behavior of apple	95 °C atmospheric pressure for 10 min, further decompression, and vacuum of 4–6 kPa for 2 h	-	The water equilibrium process prevented the collapsing of the dried apple cube after DIC and provided for fully expanded pores	[26]
	Effects of 4 different drying methods on the characteristics of cell wall polysaccharides and correlation with texture of apple chips.	200–300 kPa, 10–15 min	-	The apple chips exhibited higher crispness and better microstructure	[28,29,30,31]
	To evaluate partial air drying, DIC treatment, and/or freezing effects on apple textural characteristics	100–300 kPa; 5–45 s	200 kPa; 26 s	DIC treatment attenuates the negative impacts on textural quality of high-water content products such as apple	[32]
	To characterize the browning ratio, color changes, and polyphenol oxidase behavior of dehydrated apple slices	The samples were treated at 95 °C for 15 min, then by 5 kPa vacuum in 0.2 s.	-	Remarkable increment in the browning ratio of Air Dried-DIC textured apple slices with reduced activity of polyphenol oxidase.	[21]
Apple (*Golden delicious*)	Freezing/thawing profiles of conventionally and DIC dehydrofrozen apples	200 kPa; 25 s	-	The lower the water content, the higher the thawed apple firmness. No significant impact of freezing rate. DIC-dehydrofreezing exhibited significant reduction of thawing duration and enhanced frozen apple fruit texture.	[33]
Banana (*Musa* sp.)	Obtain and characterize DIC Treated banana flour	260–500 kPa; 12 to 48 s	500 kPa, 11 s	There were a 23% increase in effective diffusivity, 290% increase in WHC *, and 15% OHC reduction	[34]
Strawberry (*Fragaria* var. Camarosa)	To evaluate the effect of DIC on strawberry slices	200–470 kPa; 2–18 s	220–350 kPa	An increment in the expansion rate of 2.4 times was observed.	[35]
	To study the impact of DIC on antioxidant and crispiness of strawberry snacks	170–600 kPa; 10–27 s	600 kPa; 10 s	The highest crispiness: 600 kPa, 10 s The most Anthocyanin preservation: 350 kPa; 10 s.	[36,37,38]
Berry cacti (*Myrtillocactus geometrizans*)	To preserve the antioxidant capacity of berry cacti after DIC processing	100–450 kPa; 5–45 s	450 kPa; 25 s	DIC was an efficient method to dry berry cacti, comparable to freeze-drying.	[39]
Date (*Phoenix dactylifera* L.)	Obtain Zaghloul Snacks and characterize them	200–600 kPa, 9 s to 35 s	600 kPa; 22 s	146% expansion ratio achieved; 59% increase in color intensity	[40]

* WHC: water holding capacity; OHC: Oil holding capacity.

**Table 2:** DIC conditions to treat food byproducts and some key findings.

Matrix	Objective	DIC conditions: Pressure; Time	Optimal: Pressure; Time	Key Findings	References
Cactus Pear (*Opuntia ficus-indica*)	Obtain Cactus pear Snacks and characterize them	100–600 kPa; 5 s–25 s	600 kPa; 15 s	2122% expansion ratio and 83% shear force reduction. 52.1% Scavenging ability was reported, which is a 52% increase over untreated CPP	[42]
Orange (*Citrus sinensis*)	Compare the effect of DIC against hydro distillation for orange peels	600 kPa, 1 to 11 cycles and a total heating time of 30 s to 210 s	600 kPa, 11 cycles with 152 s as a total heating time	4.2% higher EO extraction yield from peels for 2.5-min treatment, instead of 4 h	[14]
Orange (*Citrus sinensis*)	Valorize Orange Industry by-products with DIC	330–600 kPa, 1–7 cycles with 20–220 s as a total heating time	490 kPa, 5 cycles, and 186 s as a total heating time	An overall increase in the compound availability was observed. E.g., a 594% increase in water diffusivity	[43]
Cranberry pomace and seeds	Improve the product′s multidimensional qualities of nutritional, hygienic, organoleptic, and convenience attributes.	200–500 kPa; 5–15 s	-	A great increase in kinetics of drying and rehydration was observed. The natural taste was preserved	[44]

**Table 3:** DIC conditions to treat vegetables and some key findings.

Matrix	Objective	DIC Conditions: Pressure; Time	Optimal Conditions: Pressure; Time	Key Findings	References
Carrots (*Daucus carota*)	Impact of DIC treatment on the expansion ratio, color, and degree of cooking of diced carrots	200–600 kPa; 2–30 s	450 kPa; 25 s	Airflow impingement just after DIC treatment as modification of DIC equipment leads to intensifying cooling of thermal sensitive foods such as carrots.	[4,13]
	Characteristics of DIC Swell-Dried, thin-layer drying of carrots compared to the traditional Hot Air Drying.	100–500 kPa, 5–55 s	440 kPa, 48 s	DIC-assisted Swell-drying can intensify the drying of carrots reducing the drying time and increasing the final quality.	[47]
	Coupling freezing and osmotic dehydration to DIC.	95 °C for 15 min, 100 kPa	-	Freezing at −18 or −40 °C modifies the carrot microstructure and thereby enhances the DIC expansion	[48,49,50]
	Effect of several DIC-pretreatments on the carrots blanching	250–550 kPa, 20 s	500 kPa, 20 s	Long freezing/thawing induced more dilation and cellular breakdown, and thus, it facilitated the diffusion of water within the cells during dehydration.	[51]
Potatoes (*Solanum tuberosum*)	Dehydration of potato slices.	200–500 kPa, 1 cycle, for 5 to 55 s	350–500 kPa, 1 cycle, 10 s	Reduction in drying time. Increase in expansion ratio.	[1,4,13]
	To confer a porous structure to potatoes by DIC texturing, thus facilitating the drying process at a lower water content	300 to 600 kPa, 1 cycle, for 20 s	600 kPa, 1 cycle, for 20 s	A lower value of water activity	[52]
Tomatoes (*Solanum lycopersicum* L.).	Effect of swell-drying of tomatoes slices on texture and rehydration	100–700 kPa; 5–45 s	400 kPa; 30 s	The firmest structure after rehydration was obtained for tomato slices and preservation of antioxidant capacity.	[13,53]
Tomato paste	To decrease drying processing time of tomato paste; while preserving the quality.	100–500 kPa; 10–50 s	300 kPa; 30 s	DIC texturing reduced drying time from 7 to 1.5 h and improved the effective water diffusivity	[54]
Onion (*Allium cepa*)	To improve the quality of processed onion in regards to color retention and maximum expansion	200 kPa; 20–30 s; and subsequently 600 kPa; 0–10 s	-	Compared to CAD, DIC swell-dried onions were threefold more expanded, with a perfectly controlled color (white, yellowish, golden, brown, even caramelized).	[4,13]
	Improve drying kinetics of onion granule powder with DIC	200–500 kPa, 1 cycle, for 5 to 55 s	350 and 500 kPa, 1 cycle, 10 s	55% reduction of drying time with 99 % reduction in microbial contamination	[27]
	Improve drying kinetics of onion with DIC	200–500 kPa, 1 cycle for 5 to 15 s	460 kPa, 1 cycle for 14 s	223% Improve in effective diffusivity and initial availability. The harsher the treatment, the more difference perceived to the original sensory profile. 3.9 log F reduction of UFC	[17]
Pepper (*Capsicum annum*)	Determine the impact of DIC on dehydration and rehydration kinetics	200–600 kPa, 1 Cycle, and 5 to 35 s	-	246% Increase in dehydration effective diffusivity and 224% in starting accessibility	[55]
Cassava roots (*Manihot esculenta*)	To study the effect of swell-drying on drying kinetics, physical product properties, and microbial decontamination of fresh cassava.	300–540 kPa; 12–48 s	400 kPa; 12 s	An increase of 6.6 and 5 times in water and oil holding capacity, respectively. An 85.7% reduction of the initial bacteria content was obtained.	[7]
Okra Pods (*Abelmoschus esculentus* (L.) Moench)	To compare swell-drying against conventional shade drying and optimize DIC texturing of green okra pods.	200–600 kPa; 40–60 s	400 kPa; 50 s	An increase of 25% and 99% of the relative expansion ratio and flavonoid content, respectively in swell-dried okra pods compared with conventional shadow dried ones	[56]
Beetroot (*Beta vulgaris* L.)	To evaluate the effect of DIC as blanching and texturing pre-treatment on the antioxidant content and antioxidant activity preservation of beetroots	100–530 kPa; 5–31 s	350 kPa; 20 s	The application of DIC as blanching and texturing pre-treatment maintained or enhanced the content of phenolic compounds and the antioxidant capacity.	[57]

**Table 4:** Summary of the DIC conditions observed in the treatment of cereals and some key findings.

Matrix	Objective	DIC Conditions: Pressure; Time	Optimal: Pressure; Time	Key Findings	References
Maize (*Zea mays*)	To compare the effects of pressure against processing time for native starch	100–500 kPa for 120 s vs. 300 kPa for 30–900 s	It depends on the objective	Both factors, pressure and time, increased starch gelatinization	[62]
	Study the effects of DIC in physicochemical Properties of Starches	100–300 kPa for 5–60 min	-	Potato starch was more prone to DIC treatment, and Rheological behavior for all the starches approximate Newtonian behavior, except waxy maize starch	[63]
	Characterize Structural modifications and thermal transitions of maize starch after DIC	100 kPa, 10–180 min. 200 kPa; 2–180 min 300 kPa; 0.5–20 min	-	Pressure influenced the viscosity positively, with time achieving a peak and then reducing viscosity	[64]
	Study the effects of DIC in physicochemical properties of starches	100–300 kPa, 600–5400 s	-	For treated starches, there was a positive correlation of pressure and time to the transition temperatures increase, gelatinization, and increased enzymatic hydrolysis sensibility	[65]
	Develop a model for water distribution change of maize starch during DIC	100 kPa; 0–60 min 200 kPa; 0–120 min 300 kPa; 0–120 min	-	As processing pressure increases, the water activity coefficient and mass transfer coefficient decreases	[66]
	Study the effects of DIC in physicochemical properties of starches	100 kPa; 90 min 200 kPa; 90 min 300 kPa; 10 min	-	Potato starch was more prone to DIC treatment, and rheological behavior for all the starches approximate Newtonian behavior, except waxy maize starch	[63]
	Compare DIC to direct vapor and reduced pressure heat treatments	100–300 kPa; 20–41 s	-	DIC created bigger granule sizes compared to the other treatments tested	[67]
Rice (*Oriza sativa*)	Study the effects of DIC on paddy rice	400–600 kPa; 15–40 s	500 kPa for 16 to 30 s	65%-time reduction in post-production cooking time	[3,68,69]
Wheat (*Triticum* spp.)	To evaluate DIC texturing on some physicochemical and nutritional properties of puffed wheat snacks products	300–500 kPa; 3–11 s	500 kPa for 7 min	A wheat grain expansion was obtained, as well as better sensorial properties	[70]

**Table 5:** Summary of the DIC conditions and some key findings in animal food.

Matrix	Objective	DIC Treatment: Pressure, Time	Optimal: Pressure, Time.	Key Findings	References
Skim milk Powder	To study the main effects of DIC operative conditions on structural, physical, and reconstitution characteristics of skim milk powder	200–1000 kPa, 2–60 s	570 kPa, 44 s	Higher powder quality with less fine powder	[71]
Leerdammer cheese from *Bos taurus* milk	Characterize cheese snacks and powdered granules	200–600 kPa, 10–30 s	550 kPa, 30 s	Up to 36 times volume increase, leading to higher porosity and lower bulk density	[72]
Ras Cheese	To study the direct expansion of cheese pieces to expand them and grinding lead to expanded granule powders.	400–500 kPa, 10–50 s	500 kPa and 10 s	DIC texturing acts on reorganizing cheese through the thermal-mechanical effects forming cavities and vacuoles and an expanded structure.	[73]
Eggs from (*Gallus gallus domesticus)*	Observe the changes in behavior of different physicochemical properties of egg products	100–700 kPa, 10–60 s	550 kPa, 33 s	The physicochemical properties are related to pressure for both egg white and yolk.	[74]
Chicken breast (*Gallus gallus domesticus*)	To develop a newly expanded texture on dried meat	400–700 kPa; 82 to 130 s	530 kPa; 110 s	An expansion ratio was obtained 15 times higher than untreated samples; the rehydration behavior was also improved.	[18,75]
Atlantic salmon (*Salmo salar*) and white tuna (*Thunnus albacore*)	To study the effect of several successive pressure-drops on fish cubes (multi-flash autovaporization)	260–540 kPa; 4–46 s		A reduction in dehydration time was observed	[76]
Shrimp *(Penaeus notialis*)	To obtain shrimp snacks and characterize them	400–700 kPa; 70–130 s	500 kPa; 70 s	More expanded with higher porosity dried material thanks to the mechanical stress caused by vapor generated within the pores	[77]

**Table 6:** Effect of DIC on the essential oil extraction, main conditions, and key findings.

Matrix	Objective	DIC Conditions: Pressure, Time; Cycles	Optimal Condition; Pressure; time, Cycles	Key Findings	References
Hyssop (*Hyssopus officinalis* L.)	Determine the optimum condition for the extraction of Hyssopus officinalis L. essential oil (EO) by instant controlled pressure-drop	100–350 kPa, 20–100 s per cycle; 1 to 12 cycles	100 kPa, 12 cycles of 100 s	DIC increases the yield and quality of essential oil, while reducing the extraction time.	[82]
*Bunium persicum* seeds	To obtain the maximum yield of essential oil	100–350 kPa, 20–60 s	350 kPa; 20 s; 9 cycles	DIC increased the yield and the quality of essential oil.	[83]
Myrtle leaves (*Myrtus communis* L.)	Compare the effect of DIC against hydro distillation for myrtle leaves	2 to 6 cycles of 100–600 kPa and total heating time of 19–221 s;	600 kPa, total heating time of 120 s; 4 cycles	DIC essential oils had a strongly spiced aroma and a particular odoriferous fragrance while being more translucent.	[84,85]
Lavender Grosso (*Lavandula intermedia* var. Grosso)	To evaluate the effect of DIC on the extraction of essential oil.	100–600 kPa, 5–60 s, 1–9 cycles	600 kPa thermal treatment time of 60 s; 2 cycles	DIC is a quick and effective process of essential oil extraction compared to steam distillation.	[6]
Rosemary leaves (*Rosemarinus officinalis* L.)	Deodorize rosemary leaves before extracting their antioxidants.	600 kPa, 6–40 s per cycle; 1 to 11 cycles	600 kPa; 16 s per cycle; 11 cycles	6.25% increase in EO extraction with improved quality through 8.44% more oxygenated compounds. The residual solid after EO extraction showed 65% improvement in antioxidant availability.	[10]
Orange peels (*Citrus sinensis*)	To extract essential citrus oil through DIC by autovaporization	600 kPa, Total time 30–210 s; 1–11 cycles	600 kPa; 11 cycles, total time of 152 s;	DIC extraction time was less than 3 min against hours for hydrodistillation.	[14]
	Apply DIC (steam explosion) to favor essential oil extraction by Clevenger apparatus.	300 to 1200 kPa, 15–480 s	800 kPa, 240 s	The swelling of the cells enabled an improved kinetic extraction in conditions of diffusivity and starting accessibility, leading to a high essential oil yield	[86]
Thyme (*Thymus capitatus*)	To use swell-drying process in the case of two different proveniences of thyme to improve texture and essential oil yield	600 kPa; 20–60 s; 1–7 cycles	10 s; 4 cycles	Swell-drying increases up to 10 times the effective diffusivity and twice the starting accessibility better than CAD. A relative expansion ratio was almost two.	[87]

**Table 7:** Antioxidant extraction as modified by DIC treatment.

Matrix	Objective	DIC Conditions: Pressure; Time	Optimum Condition: Pressure; Time	Key Findings	References
Orange peels (*Citrus sinensis*)	Valorize orange peels through phenol extraction.	300–600 kPa, total time of 20–220 s for 1–7 cycles	490 kPa, for 186 s in 5 cycles	Increased effective diffusivity and starting accessibility, higher extraction yield in total phenol.	[43]
Hibiscus Flower (*Hibiscus sabdariffa*)	Effect of DIC on anthocyanin extraction	70–200 kPa, 5–30 s	180 kPa, 18 s	135% improvement in Total Monomeric Anthocyanin extraction and a 10–45% improvement in effective diffusivity.	[93]
Pomegranate peel (*Punica granatum* L.)	Effect of DIC texturing on total phenolic compounds extraction	100–300 kPa, 10–60 s for 1–5 cycles.	300 kPa, 60 s in 1 cycle.	Expanded material has lower mass transfer resistance and consequently enhances the extraction efficiency.	[94]
Olive leaves (*Olea europaea* L.)	Obtain solvent extraction kinetics of total phenolic content in olive leaves	100–700 kPa; 10–30 s; 1–3 cycles	100 kPa, 11 s; 1 cycle	176%, 103%, and 62% improvement in yields, starting accessibility, and overall diffusivity of total phenol extraction	[95,96,97]
Chokecherry (*Prunus virginiana* L.)	Effect of drying methods on the antioxidant content and activity of chokecherry fruit	240–460 kPa, 10–22 s	270 kPa, 20 s	DIC treatment leads to better preservation of antioxidant compounds at room temperature.	[98]
Stalk grape (*Vitis vinifera. L*)	Impact of DIC texturing on the extraction of phenolic compounds from stalk powder	300 kPa, 50 s	-	DIC treatment increased the availability of gallic acid, quercetin, ellagic acid, and resveratrol	[99]
Tea (*Camellia sinensis* cv. Jiukeng)	To induce textural changes by DIC	400 kPa, 0.1 s	-	The brewing of DIC processed tea can be performed in ambient temperature water.	[100]

**Table 8:** Extraction of vegetable oil from raw materials treated by DIC.

Matrix	Objective	DIC Conditions: Pressure; Time	Optimum Condition: Pressure; Time	Key Findings	References
Rapeseed seeds (*Brassica napus* L.)	Modeling of solvent extraction kinetics to identify the fundamental impact of DIC treatment and grinding of rapeseed seeds	200–700 kPa; 20–120 s	510 kPa, 50 s	All DIC tested conditions improve oil extraction compared to untreated seeds.	[5]
	Enhancing oil extraction yield from raw material either by pressure or solvent extraction	100–700 kPa; 20 to 120 s	580 kPa; 86 s	DIC-texturing technology intensified both rapeseed solvent (hexane) and pressing oil extractions	[105]
Soybean (*Glicine max*)	Impact of the different mechanical pre-treatments and thermal/mechanical texturing ways on solvent extraction of soybeans.	200–700 kPa; 20 to 120 s	490 Pa; 96 s,	DIC pre-treatment increases oil yield while decreasing the extraction time without affecting the oil quality	[106]
Safflower r (*Carthamus tinctorius* L.)	DIC texturing affects the mass transfer and increases oil extraction from safflower seeds	100–350 kPa; 20–120 s; 1–5 cycles	240 kPa, 20 s, 5 cycles	DIC-treated samples had improved mass transfer and oil yield.	[107]
Sunflower seeds (*Helianthus annuus* L.)	DIC treatment to enhance cold-press oil extraction	200–700 kPa; 15–75 s:	500 kPa; 50 s	There was higher oil availability, increasing yields of the cold-press oil extraction.	[108]
Date palm (*Phoenix dactylifera* L)	To use DIC as a pretreatment to extract oil by supercritical fluids.	100/700 kPa; 20–60 s	619 kPa; 54 s	The higher the DIC pressure and treatment time, the higher the seed porosity. By coupling DIC to supercritical carbon dioxide extraction, the oil extraction from date seeds was intensified.	[109]

**Table 9:** DIC treatment of legumes to reduce allergenicity.

Matrix	Objective	DIC Conditions: Pressure; Time	Optimum Condition	Key Findings	References
Peanut (*Arachis hypogaea* L.) Lentil (*Lens culinaris*) Chickpea (*Cicer arietinum*) Soybean (*Glycine max*)	Impact of DIC treatment on peanut, lentil, chickpea, and soybean IgE antibody reactivity	300–800 kPa, 33–180 s	600 kPa for 180 s	DIC treatment produces a reduction in the overall in-vitro IgE binding of peanut, lentil, and chickpea and a drastic reduction in soybean immunoreactivity	[11,120]
Lupins *(Lupinus albus* var Multolupa)	Effect of DIC on in vitro lupin allergenicity	300, 450, and 800 kPa for 60, 120 and 180 s	600 kPa, 180 s	The combination of heat and steam provided by DIC eliminated the allergenicity of lupin	[115]
Pistachios (*Pistacia vera*) and cashew nut (*Anacardium occidentale*)	Effect of DIC on the allergenicity of pistachios and cashew nut proteins	300–700 kPa, 43–120 s	700 kPa, 120 s	Although DIC treatments reduced the allergenicity of pistachios and cashew nuts, it was less effective than autoclave treatments	[116]
Wheat gluten (*Triticum aestivum* L.)	Effect of DIC on chemical, functional, and immunological properties of wheat gluten powder	100–165 °C; 20–60 s	165 °C, 60 s	DIC treatment-induced formation of β-sheet, making gluten structure more rigid and modifying its functional properties. The immunoreactivity of gluten still depends on the patient; however, it was increased.	[121]

**Table 10:** Evaluation of DIC treatment on the content of non-nutritional factors in legumes.

Matrix	Objective	DIC Conditions: Pressure; Time	Optimal Condition: Pressure; Time	Key Findings	References
Soybean (*Glycine max*); Lupins *(Lupinus albus* var Multolupa); Lentil (*Lens culinaris*); Chickpea (*Cicer arietinum*); Peanut (*Arachis hypogaea* L.)	To study the effect of different conditions of pressure and time applied during DIC treatment to legumes, to reduce antinutritional factors.	300–600 kPa; 60 s and 180 s	600 kPa; 60 s	The DIC treatment increases the availability of soluble sugars but decreases phytates, lectin content, and trypsin inhibitors.	[124]
Sarphonka *(Tephrosia Purpurea)*	Compare the effect of DIC on oligosaccharides extractability on *Tephrosia purpurea* seeds	200–600 kPa, 30–240 s	520 kPa; 197 s	Improvement in the extraction of ciceritol in 150% and 173% for stachyose, in 1 h instead of four hours.	[125]
Rapeseed (*Brassica napus* L.)	To detoxify rapeseed flour by reducing glucosinolate content	160–700 kPa; 6–74 s	700 kPa; 74 s	A reduction of 40% in glucosinolate content is obtained in 60 s. The lowest level of glucosinolate corresponds to the highest pressure and time treatment.	[126]
Black bean (*Phaseolus vulgaris* L.)	Reduce Anti-nutritional factors in seeds and sprouts	100–300 kPa; 10–80 s	It depends on the factor to optimize	DIC reduced the availability of Trypsin inhibitors, Saponins and Tannins	[12]
